# Single cell atlas reveals multilayered metabolic heterogeneity across tumour types

**DOI:** 10.1016/j.ebiom.2024.105389

**Published:** 2024-10-10

**Authors:** Zhe Zhou, Di Dong, Yuyao Yuan, Juan Luo, Xiao-Ding Liu, Long-Yun Chen, Guangxi Wang, Yuxin Yin

**Affiliations:** a Institute of Systems Biomedicine, Department of Pathology, School of Basic Medical Sciences, Peking University Health Science Centre and School of Life Sciences, Peking University, Beijing 100191, China; b Institute of Precision Medicine, Peking University Shenzhen Hospital, Shenzhen 518036, China; c Research Centre for Molecular Pathology, Department of Pathology, Peking Union Medical College Hospital, Chinese Academy of Medical Sciences and Peking Union Medical College, Beijing 100032, China

**Keywords:** Pan-cancer analysis, Cancer metabolism, Metabolic heterogeneity, scRNA-seq, Chemotherapy resistance

## Abstract

**Background:**

Metabolic reprogramming plays a pivotal role in cancer progression, contributing to substantial intratumour heterogeneity and influencing tumour behaviour. However, a systematic characterization of metabolic heterogeneity across multiple cancer types at the single-cell level remains limited.

**Methods:**

We integrated 296 tumour and normal samples spanning six common cancer types to construct a single-cell compendium of metabolic gene expression profiles and identify cell type-specific metabolic properties and reprogramming patterns. A computational approach based on non-negative matrix factorization (NMF) was utilised to identify metabolic meta-programs (MMPs) showing intratumour heterogeneity. In-vitro cell experiments were conducted to confirm the associations between MMPs and chemotherapy resistance, as well as the function of key metabolic regulators. Survival analyses were performed to assess clinical relevance of cellular metabolic properties.

**Findings:**

Our analysis revealed shared glycolysis upregulation and divergent regulation of citric acid cycle across different cell types. In malignant cells, we identified a colorectal cancer-specific MMP associated with resistance to the cuproptosis inducer elesclomol, validated through in-vitro cell experiments. Furthermore, our findings enabled the stratification of patients into distinct prognostic subtypes based on metabolic properties of specific cell types, such as myeloid cells.

**Interpretation:**

This study presents a nuanced understanding of multilayered metabolic heterogeneity, offering valuable insights into potential personalized therapies targeting tumour metabolism.

**Funding:**

10.13039/501100012166National Key Research and Development Program of China (2021YFA1300601). 10.13039/501100001809National Natural Science Foundation of China (key grants 82030081 and 81874235). The Shenzhen High-level Hospital Construction Fund and Shenzhen Basic Research Key Project (JCYJ20220818102811024). The Lam Chung Nin Foundation for Systems Biomedicine.

**Research in context**

**Evidence before this study**

We searched PubMed with the keywords “single cell”, (“cancer” or “pan-cancer”), metabolic (“heterogeneity” or “landscape”) for articles published up to April 23, 2024, with no language restrictions. We found that previous studies have primarily focused on specific cell types or cancer types and have often utilized low-throughput methods, limiting analysis to a subset of metabolic enzymes. This underscores the importance of conducting comprehensive investigations into single-cell metabolic heterogeneity across diverse cancer types using high-throughput approaches.

**Added value of this study**

In this study, by compiling both new generated and publicly available datasets, we constructed a single-cell compendium of metabolic gene expression profiles from 296 tumour and normal samples spanning six common cancer types. We systematically compared metabolic properties and reprogramming patterns between major cell types in the tumour microenvironment (TME), revealing commonalities in elevated glycolysis but significant difference in other metabolic processes. Through the identification of metabolic meta-programs (MMPs) characterizing intratumour heterogeneity, we explored associations with chemotherapy resistance, metabolic regulation, and functional differentiation of cells in TME. Our approach enabled the stratification of patients into distinct prognostic subtypes based on specific cell types, offering new insights into precision medicine and personalized treatment strategies.

**Implications of all the available evidence**

Metabolic adaptations of tumours exhibited complex inter- and intratumour heterogeneity, posing challenges for effective cancer treatment. Our findings provide valuable insights into metabolic heterogeneity, paving the way for precision medicine approaches tailored to individual patients. For example, the identification of colorectal cancer cells with higher MMP7 scores showing resistance to cuproptosis inducer elesclomol highlights the potential for targeted therapies. Our new metabolic subtyping strategy offers insights into innovative treatments. Furthermore, our study establishes an easily extendable framework for understanding single cell level metabolic heterogeneity across various cancer types, with implications for advancing translational research and clinical practice.

## Introduction

Cancer cells undergo extensive metabolic remodelling to fulfil the energy and biosynthetic demands crucial for rapid proliferation, a well-established hallmark of cancer known as metabolic reprogramming.^1^ Key metabolic traits of cancer cells encompass a predilection for aerobic glycolysis, reliance on glutamine, heightened macromolecular synthesis and maintenance of redox homeostasis. The intricacies of these adaptations stem from a myriad of intrinsic and extrinsic cues,^2^^,^^3^ including genomic alterations, tissue-specific characteristics, elements within the tumour microenvironment (TME), and individual-level metabolic variations. The resulting metabolic heterogeneity across cancer types and individuals poses challenge for developing therapies targeting metabolic vulnerabilities.

The tumour functions as a complex ecosystem that harbours a variety of cell types. The intricate crosstalk among these cell types profoundly shapes cancer progression, and their unique metabolic demands are closely linked to specific functions.^4^^,^^5^ Although studies on metabolic reprogramming have yielded invaluable insights into cancer biology, the majority were conducted on cultured cells, model organisms, or bulk tumours. To unravel intratumour metabolic heterogeneity, a closer examination of single-cell metabolic configurations within the complex TME is imperative. However, limitations in single-cell metabolomics, including the necessity for specialized mass spectrometry (MS) instrumentation, challenges in analysing densely cultured cells, and the absence of suitable computational methods for downstream analysis, hinder comprehensive exploration.^6^ Considerable effort has been expended to investigate single-cell metabolic profiles via multiplexed mass cytometry^7^ and in situ enzyme histochemistry.^8^ However, these approaches are relatively low throughput, typically being limited to several metabolic enzymes.

A holistic understanding of cellular metabolism necessitates a meticulous examination of both metabolite concentrations and their conversion fluxes, which is difficult to conduct in diverse human tissues. Notwithstanding, global gene expression levels offer a measurable molecular dimension bridging oncogenic drivers to metabolic phenotypes. Several studies have revealed widespread transcriptional dysregulations of metabolic genes and their associations with patient prognosis through the analysis of large-scale gene expression data.^9^, ^10^, ^11^ With the advent of single-cell RNA sequencing (scRNA-seq), some pioneering studies have investigated cellular metabolic heterogeneity in one or two isolated cancer types.^12^^,^^13^ Computational methods for inferring metabolic flow from bulk and scRNA-seq data have been developed, providing valuable insights into the tumour metabolic microenvironment.^14^^,^^15^ Nonetheless, it is not clear whether such findings can be extended to other cancer types. Moreover, metabolic reprogramming of distinct cell types in TME relative to cells from normal tissues have not yet been examined. A systematic investigation of the intratumour metabolic heterogeneity of various cell types across a wider array of cancer types is urgently needed.

In this study, we constructed a metabolic gene expression atlas using scRNA-seq datasets and investigated multilayered metabolic heterogeneity across cell types, single cells within specific tumours, as well as individuals. Our findings provide a vital resource for comprehending the nuanced metabolic heterogeneity present in multiple cancer types.

## Methods

### Data curation

A total of 10 scRNA-seq datasets including 197 tumour samples and 99 normal samples were curated. Among these, BRCA_valid2 and LUAD_valid2 datasets include data generated by our laboratory and collaborators, while the remaining datasets were sourced from the literature (Supplementary Table S1). For the BRCA_valid2 dataset, newly generated data include samples from estrogen receptor-positive breast cancer (ERBC) and paired normal tissues. The LUAD_valid2 dataset consist of tumour samples generated by our lab and analysed in a previous study, with normal samples collected from literature. Our data was not deliberately enriched for a particular sex in none of the studies except for cancer types which are sex specific (e.g., breast cancer or prostate cancer), and this data was self-reported by study participants.

### scRNA-seq data generation

For data generated by our lab and collaborators, samples were collected at Peking University People’s Hospital. This study was approved by the Ethics Committee Board of Peking University People’s Hospital (2021PHB387-001), and informed consent was provided by all patients. Preparation of single-cell suspensions was described previously.^16^ Briefly, tissues were transported in ice-cold H1640 (Gibco, Life Technology) immediately after surgical resection. Then, the tissues were minced into ∼1 mm^3^ pieces, digested with 0.25% trypsin (Gibco, Life Technology), and transferred to 10 mL digestion medium containing collagenase IV (100 U/mL, Gibco, Life Technology) and dispase (0.6 U/mL, Gibco, Life Technology). The digested tissues were successively filtered through 70-μm and 40-μm nylon mesh and treated with ice-cold red blood cell lysis solution (Solarbio). Finally, the pelleted cells were suspended in 1 mL of Dulbecco’s phosphate buffered saline (DPBS, Solarbio), and the concentrations of live cells and clumped cells were determined using an automated cell counter (Countstar). The cell suspension was loaded onto the chromium single cell controller approximately 10,000 cells/chip position using the Single Cell 3’ Library and Gel Bead Kit V2 (10X Genomics) and Chromium Single Cell A Chip Kit (10X Genomics) according to the manufacturer’s instructions. All the subsequent steps were performed following the standard manufacturer’s protocols. Purified libraries were analysed by an Illumina HiSeq X Ten sequencer with 150-bp paired-end reads.

### scRNA-seq data preprocessing

For data generated by our lab and collaborators, Cell Ranger (version 3.0, 10x Genomics Inc) was used for sequencing reads mapping against the GRCh38 human reference genome and unique molecular identifiers (UMIs) counting. For previously published scRNA-seq data, count data were collected from original publications (Supplementary Table S1). Only samples of primary cancer and normal controls were kept, and cells with fewer than 200 detected genes or 500 UMI counts were filtered out. No more than 10% mitochondrial reads were generally allowed per cell, although the upper limit was increased as high as 40% for a small number of libraries according to its distribution.^17^ DoubletFinder^18^ was applied to each sequencing library to remove potential doublets. Finally, all datasets were downsampled to no more than 100,000 cells to reduce the computational burden.

### Cell type identification

Uniform pipelines were performed on all datasets to identify the main cell lineages, although cell annotation tables of published data were obtained as references. Seurat^19^ (v4.3.0) was used to identify top 2000 highly variable genes, perform dimensional reduction and cluster cells into groups, which were then annotated using canonical markers of the main cell lineages (Supplementary Table S2).

To distinguish malignant cells from non-malignant epithelium, inferCNV (https://github.com/broadinstitute/inferCNV) was used to estimate initial copy number variations (CNVs) for BRCA, CRC, LUAD and PAAD. The CNV score of each cell was calculated as the quadratic sum of the inferred CNV values. For cancers with low CNV signals (PRAD and STAD), we used the algorithm described by Zhang et al.^20^ to identify malignant cells. Briefly, differentially expressed genes between tumour and normal samples from The Cancer Genome Atlas (TCGA) database were first calculated using the limma^21^ package. Then, each epithelial cell was assigned an initial malignant/non-malignant score based on the top 50 highly expressed genes. Putative malignant and non-malignant epithelial cells were defined based on the two scores using the k-means clustering algorithm. The initial recognition derived from the TCGA bulk tissues is biased due to the inclusion of non-epithelial cells. Thus, we next generated differentially expressed genes between putative malignant and non-malignant epithelial cells, calculated new malignant/non-malignant scores and reclassified epithelial cells. These processes were repeated iteratively until the misclassification rate (compared to the previous round) was less than 0.001.

CD4^+^ (CD3^+^CD4^+^CD8^-^) and CD8^+^ (CD3^+^ CD4^-^ CD8^+^) T cells were isolated based on the average expression of CD3 genes (*CD3D*, *CD3E*, *CD3G*), *CD4* and CD8 genes (*CD8A*, *CD8B*). Briefly, cells with CD3 > 0, CD4 > 0 and CD8 = 0 were assigned as CD4^+^; cells with CD3 > 0, CD4 = 0 and CD8 > 0 were assigned as CD8^+^; other T cells were unassigned.

### Pathway scoring

Metabolic genes and pathways were manually curated based on a previous study^9^ and out-of-date gene symbols were updated (Supplementary Table S3). Gene sets of Gene Ontology (C5.GOBP) and Hallmark (H) were downloaded from MsigDB (https://www.gsea-msigdb.org/gsea/msigdb/), and cancer hallmarks were manually curated from Hallmark gene sets. Signatures of T and B cell subtypes were defined by Zheng et al.^22^ and Ma et al.,^23^ and the top 50 genes of each subtype were used. Signatures of the functional phenotype of macrophages were obtained from a previous study.^24^

Pathway scores of metabolic pathways, regulons, cancer hallmarks and immune signatures for single cells were calculated using the command *aucell* of pyscenic. Pathway scores for bulk, pseudo-bulk and cell type specific pseudo-bulk samples were calculated using GSVA.^25^

### Identification of cell type-specific metabolic signatures and reprogramming patterns

Cell type-specific metabolic signatures and reprogramming patterns were identified using the *FindMarkers* function in Seurat with the default Wilcoxon rank sum test, and *P* values were adjusted using the Benjamini & Hochberg (BH) method. Significant genes were defined as those with log_2_ fold change >0.1, false discovery rate (FDR) < 0.01 and percent of expressed cells in either population >10%. For pathways and regulons, the log_2_ fold change threshold was set as 0.01.

### Cell lineage prediction using metabolic gene expression

For each dataset, samples were split randomly into two groups with equal size and 10,000 cells were subsampled from each group to form the training and test sets, respectively. Then L1-regularized linear regression models were trained on the training sets using the glmnet R package with 10-fold cross validation, and AUC values were calculated on test sets for performance evaluation.

### Quantification of cell-to-cell metabolic similarity

Due to the high dropout rates in scRNA-seq data, the correlation coefficient of metabolic gene expression was significantly correlated with the number of expressed metabolic genes in cells. Thus, principal component analysis (PCA) was performed on metabolic gene expression profiles, and the Spearman correlation coefficient of the first 30 PCs was calculated as cell-to-cell metabolic similarity.

### Analysis of mass cytometry data

Mass cytometry data of CRC were collected from a previous study^7^ and clustered using the FlowSOM^26^ R package and the indicated input channels. The resulting clusters were manually annotated as the main cell lineages based on their lineage markers. Uniform manifold approximation and projection (UMAP) embeddings were calculated using the R uwot implementation with the following parameters: n_neighbors = 15 and min_dist = 0.02.

### Identification of metabolic meta-programs (MMPs)

MMPs were defined according to an algorithm described by Gavish et al.^27^ with minor modification, with the following steps employed.

#### Data preprocessing

Samples with fewer than 20 cells were excluded for each cell type. The expression matrix of each sample was normalized using Seurat *NormalizeData* function and top 500 metabolic genes with highest mean expression were retained. Gene expression values were scaled across cells, and negative values in each scaled matrix were set to zero.

#### Identifying NMF programs

Non-negative matrix factorization (NMF) was performed on each sample separately to capture the metabolic heterogeneity within each tumour. Multiple *K* parameters (ranging from 4 to 9) were set, resulting in 39 programs for each tumour. Each program was summarized by top 30 genes based on NMF coefficients. Robust NMF programs were defined as below: (1) robust within the sample (at least 70% gene overlap with NMF programs identified by different *K* value); (2) robust across tumours (at least 20% gene overlap with NMF programs from other tumours); (3) non-redundant within the tumour (within each tumour, NMF programs were sorted in descending order by max gene overlap with NMF programs from other tumours; then programs were selected from top to bottom, and once an program was selected, any other programs with more than 20% gene overlap with the selected one was removed).

#### Defining MMPs

Robust NMF programs from all tumours were clustered using a custom approach. In brief, programs were first sorted in descending order by number of similar programs with more than 20% gene overlap (must be programs from other tumours due to non-redundancy within the tumour). The top program was selected as founder of a new cluster, and MMP was initialized as the genes in the program. Next, the program with the highest gene overlap with MMP was added and the MMP was updated as top 30 genes with the most occurrence in all included programs. This process was repeated until no programs have more than 20% gene overlap with this MMP and the resulted MMP was recorded. An attempt to find and extend a new cluster was made as above in the remaining programs until the number of similar programs less than 5 cases. When clustering is done, MMPs with less than 10 programs were removed and the remaining MMPs were annotated according to enriched metabolic pathways.

### MMP abundance assessment

The observed number of MMP-related programs for each cancer type was counted as it is. The expected number of MMP-related programs for each cancer types was calculated as $\frac{N_{MMP}\times N_{cancer}}{N_{robust}}$, where N_MMP_ is the number of programs in a MMP, N_cancer_ is the number of robust programs in a caner type, and N_robust_ is the number of all robust programs. Then abundance was calculated as $\log_{2}\frac{observed\;+\;1}{expeted\;+\;1}$, and the Bonferroni-adjusted *P* value was defined by hypergeometric test. Finally, abundance was stratified as: high (observed >10 or abundance >1; if adjusted *P* value < 0.05 then high and significant), medium (2 ≤ observed ≤10 or 0 < abundance ≤1), low (observed = 1 and −1.5 < abundance ≤0), absent (others).

### Metabolic regulons and MMP regulators

The python implementation of the SCENIC^28^ algorithm (pyscenic) was used to infer the gene regulatory network. The transcription factor list and annotation files used were downloaded from cisTarget (https://resources.aertslab.org/cistarget). Transcription factors and their metabolic targets were kept to construct metabolic regulons, and regulons with fewer than five targets were removed.

To identify MMP regulators, we calculated both the Pearson and Spearman correlation between MMP scores and regulon scores within each tumour. For each combination of dataset and MMP, the Pearson correlation coefficients were then averaged between tumours, and significance was defined by one-sided t-test and adjusted by BH correction. Regulons with adjusted *P* value (both Pearson and Spearman) < 0.05 and absolute mean Pearson correlation coefficient >0.4 were kept. If more than three regulons exist, then only top three were retained. Finally, regulons for all combinations of datasets and MMPs were combined for visualization.

### Spatial transcriptomics prep, sequencing and analysis

Formalin fixed paraffin embedded (FFPE) tissues were sectioned and placed on a Visium Spatial Gene Expression Slide. Initially, tissues sections underwent deparaffinization, staining and decrosslinking as directed by Visium Spatial Protocol CG000409 Rev B. Subsequent steps, including probe hybridization, ligation, release, extension and library construction, were conducted in accordance with Visium Spatial Protocol CG000407 Rev C. Briefly, Human whole transcriptome probed panels were introduced to the tissue, hybridized to their gene targets and subsequent intermolecular ligation. Upon RNase treatment and permeabilization, the resulting ligation products were released from the tissue and captured by primers on a specific spot share a common spatial Barcode. The libraries were generated from the probes and subjected to sequencing by an Illumina HiSeq X Ten sequencer, with all kits employed in the process sourced from 10X Genomics.

Then feature-barcode matrix and associated H&E image for each sample were obtained using Space Ranger (v1.3.1) with GRCh38 genome reference 2020-A. And spots were manually annotated by a pathologist using Loupe Browser 5.0. Finally, Seurat (v4.3.0) was used for visualization.

### Correlation between pathway or MMP scores with various factors

For correlation of pathway scores with clinical metrics, the individual CNV score was calculated as the average of all malignant cells from specific individual. Metrics including age, sex, clinical stage and CNV score were tested for correlation with metabolic pathway scores. For each of those metrics, a linear model was fitted using the R function *lm*. The adjusted R^2^ was interpreted as the proportion of variance explained (PVE). The *P* value of the F test by function *anova* and adjusted *P* value using the BH method were reported.

The Pearson correlations between MMP scores and other gene sets (cancer hallmarks, immune signatures and meta-programs) were calculated within each tumour, similar to correlations between MMPs score and regulon scores, and then averaged within each dataset or across all tumours. The significance was defined by one-sided t-test and adjusted by BH correction. Correlations with adjusted *P* value (both Pearson and Spearman) < 0.05 and absolute mean Pearson correlation coefficient >0.1 were assigned as significant.

### IHC staining

Demographic information of samples for IHC staining was provided in Supplementary Table S1. Tissue sections underwent deparaffinization and rehydration, followed by the blocking of endogenous peroxidase activity using 3% (v/v) hydrogen peroxide in methanol. Antigen retrieval was achieved with 1 mM EDTA buffer (pH 7.4) in a microwave oven. Sections were then incubated in 3% BSA for 30 min to prevent nonspecific staining. Primary antibodies against panCK (1:500, Servicebio, GB122053) and SOD1 (1:500, Santa Cruz, sc-17767, RRID AB_628301) were applied and allowed to incubate overnight at 4 °C. Fluorescent secondary antibodies (Servicebio, G1222 and G1223) were used for staining, with a 50-min incubation at room temperature. Whole-slide imaging was performed using the Pannoramic MIDI scanner (3DHISTECH). Quantification of fluorescence intensity was performed using ImageJ.

### CCLE data analysis

Gene expression data were downloaded from the Cancer Cell Line Encyclopedia (CCLE) website (https://portals.broadinstitute.org/ccle). Metabolomics data and drug response data were obtained from previous studies.^29^^,^^30^

For analysis between breast cancer subtype, only cell lines derived from primary tumours of either estrogen receptor-positive breast cancer (ERBC) or triple-negative breast cancer (TNBC) were kept, and pathway scores were calculated using GSVA. Significant metabolic pathways (FDR <0.05) and metabolites (*P* value < 0.05) between BRCA subtypes were defined via unpaired two-tailed t test.

For drug sensitivity analysis, MMP scores for cell lines were defined using GSVA. Pearson correlations were calculated between MMP scores and IC50 values for each cancer type, and *P* values for correlation test were adjusted using the BH method. Correlations with adjusted *P* value (both Pearson and Spearman) < 0.05 and absolute mean Pearson correlation coefficient >0.3 were assigned as significant.

### Cell culture and establishment of stable cell lines

NCI-H1299 (RRID CVVL_0060), PANC-1 (RRID CVCL_0480), HCT116 (RRID CVCL_0291) and HEK293T (RRID CVCL_0063) cell lines were purchased from the American Type Culture Collection, authenticated by STR locus analysis and tested for mycoplasma contamination. All cell lines used in this study were maintained in DMEM (Corning), supplemented with 10% FBS (PAN) in a 37 °C incubator with 5% (v/v) CO_2_.

The shRNA targeting sequences for human FOSL1 were synthesized and cloned into the pLKO.1-TRC cloning vector (Addgene, RRID Addgene_10878). Then the vectors were co-transfected with PMD2.G (Addgene, RRID Addgene_12259) and psPAX2 (Addgene, RRID Addgene_12260) into HEK293T cells at a ratio of 4:1:3 for 48 h and the cell culture media with virus was collected for transfection. PANC-1 and H1299 cells were transfected with virus supernatant for 12 h shRNA positive cells were selected by puromycin at a dose of 2 μg/mL for 4 days. The efficiency of shRNA was measured by reverse-transcription quantitative polymerase chain reaction (RT-qPCR).

### Drug sensitivity assays

Elesclomol (TargetMol, T6170) was dissolved in dimethyl sulfoxide (DMSO) to prepare a stock solution. The impact of elesclomol on cell viability was assessed through a colorimetric assay utilizing Cell Counting kit-8 (CCK8, Beyotime, C0038). HCT116 cells were seeded in 96-well plates at a density of 2 × 10^3^ cells per well and allowed to adhere for 12 h. Subsequently, cells were treated with varying concentrations of elesclomol (ranging from 0 to 100 μM) or DMSO for a 24-h period. The absorbance at 450 nm was measured using a spectrophotometer, and the experiment was conducted in six replicates. Cell viability curves corresponding to different concentrations were generated using Prism GraphPad software v9.0.2. The data is presented as the mean ± SEM.

### RT-qPCR

RNA was isolated from cultured cells using TRIzol (Invitrogen, 10296010) according to the manufacturer’s protocol. RNA was reverse transcribed using a commercial kit (TransGen Biotech, AE341-02), and RT-qPCR was run in an ABI 7500 Real-Time PCR System using TransStart® Green qPCR SuperMix (TransGen Biotech, AQ101) and gene-specific primers. For quantitative analysis, samples were normalized using ACTB with the delta CT method.

### Bulk RNA-seq

Total RNA was isolated from cultured cells using TRIzol (Invitrogen, 10296010). After quality check, samples were sent to library preparation using the NEBNext Ultra RNA Library Prep Kit for Illumina (#E7530L, NEB, USA). High-throughput sequencing was performed using the Illumina HiSeq X Ten platform with 150 bp paired-end reads according to the manufacturer’s instructions. FastQC was used for quality control of the sequencing data. Reads were aligned to the human (GRCh38) reference genome using STAR^31^ (v2.7.2b) and assigned to genes using featureCounts^32^ (v2.0.1). Differentially expressed genes between experimental groups were identified using DESeq2^33^ (v1.38.3).

### Sample preparation and data analysis for metabolomics study

Cells were harvested and mixed with methanol. After centrifugation at 13,000 rpm at 4 °C for 15 min, the supernatant was recovered, and metabolites levels were measured with ultrahigh-performance liquid chromatography-tandem mass spectroscopy (MS/MS). Raw data were extracted, peak-identified and quality-control processed using MSDIAL software. PCA was performed to reduce the dimension of the data. The pathway analysis using MetaboAnalyst (http://www.metaboanalyst.ca) with the default setting was performed for the metabolites significantly changed (unpaired two-tailed Student’s t test, *P* < 0.05) in knockdown cell lines compared to controls.

### Co-occurrence of MMPs of different cell types

The fraction of cells with MMP score >0.5 in each tumour was calculated and the resulted value were scaled within each dataset. Then Pearson correlations were calculated between MMPs of all cell types and only positive correlations with *P* value < 10^−5^ were kept for plot.

### Clustering of metabolic pathways and cell type specific pseudo-bulk samples

Consensus clustering (consensusClusterPlus package in R) was used to determine the optimal number of clusters using 1000 iterations and resampling of 80%.

### Survival analysis of TCGA data

The TCGA Toil re-computed expression data were downloaded from the UCSC Xena website (https://xenabrowser.net/). Clinical information of TCGA patients was obtained from PanCanAtlas (https://gdc.cancer.gov/about-data/publications/pancanatlas). Samples of colon adenocarcinoma and rectum adenocarcinoma were combined as CRC, lung cancer (LC) samples were also combined, and PRAD was not included due to lack of clinical stage data. We performed Cox regression with age, sex, and clinical stage as covariates on TCGA data of each cancer type. Then the per-cancer-type models were combined into a pan-cancer model by meta-analysis using a random effect model implemented using R package meta. Kaplan–Meier curve was plotted to show the survival difference using R package survival and survminer.

### Statistics

Statistical analysis was indicated in the Figure legends or specific Methods sections.

### Data and code availability

Bulk RNA-seq data can be obtained from Gene Expression Omnibus (GSE230729). Accession number of all scRNA-seq datasets were described in Supplementary Table S1. ST data from our unpublished study are available from the corresponding author upon reasonable request, and data from the previous study^34^ can be found on the HTAN Data Coordinating Centre Data Portal at the National Cancer Institute: https://data.humantumoratlas.org/ (under the HTAN WUSTL Atlas). The authors declare that all the other data supporting the findings of this study are available within the article and its Supplementary information files and from the corresponding author on reasonable request.

Our analysis code has been uploaded into the GitHub (https://github.com/ZedekiahZhou/PanCan_scMetab). All software and algorithms used in this study are publicly available and are listed in the Methods section.

### Role of the funding source

The funders did not play any role in the study design, data collection, management, analysis, interpretation, review, approval of the manuscript, or the decision to submit the manuscript for publication. All authors were not precluded from accessing the data in the study, and they accept responsibility to submit for publication.

## Results

### Construction of single-cell transcriptome atlas of metabolic heterogeneity

**Fig. 1:**
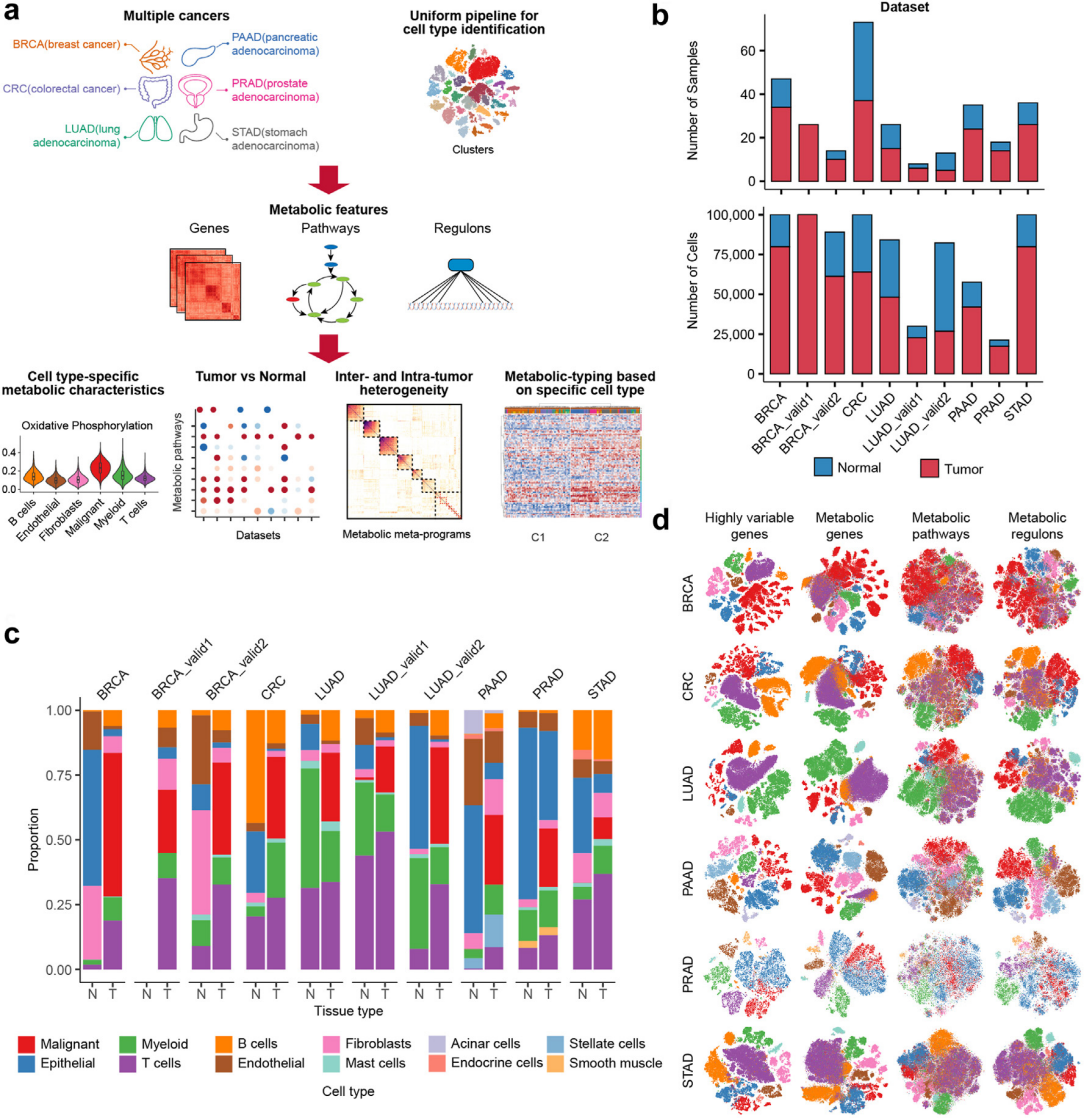
Pan-cancer landscape of metabolic gene expression at single-cell resolution. **(a)** Schematics of the computational framework. **(b)** Bar plots showing the number of samples and cells derived from tumour and normal tissues in each dataset. **(c)** Bar plots showing the cell type composition of tumour and normal tissues in each dataset. Of note, no normal tissues were collected in the original publication of the BRCA_valid1 dataset. N, normal tissues; T, tumours. **(d)** t-distributed stochastic neighbour embedding (t-SNE) visualization of the main cell lineages for the main datasets based on highly variable genes, metabolic genes, pathways and regulons, respectively.

We devised a computational framework to jointly explore the metabolic gene expression profiles in diverse solid tumours at single-cell resolution (Fig. 1a). In brief, we curated and annotated 10 scRNA-seq datasets covering six common cancer types (BRCA, CRC, LUAD, PAAD, PRAD and STAD). After rigorous quality-control filtering, the datasets comprised 764,432 cells from 296 samples, integrating both tumour and normal tissues (Fig. 1b). While most samples were from public datasets, BCRA and LUAD incorporated additional data from samples collected by our laboratory and collaborators (Supplementary Table S1). Utilizing uniform pipelines, we processed all datasets, identifying 12 main cell lineages, seven of which were shared across all datasets (Fig. 1c; Supplementary Fig. S1 and Table S2). Malignant cells and non-malignant epithelium were distinguished by copy number variations (CNV) or a score-based method (Supplementary Fig. S2). Subsequently, a curated metabolic gene and pathway signature (Supplementary Table S3) was collected, and metabolic regulons (transcriptional regulators and their metabolic target genes) were reconstructed via SCENIC^28^ analysis. This process culminated in the construction of a single-cell transcriptome atlas of metabolic heterogeneity, serving as the foundation for downstream analysis.

To assess the overall characteristics of metabolic gene expression, we investigated the number of expressed metabolic genes, the UMI counts, and the percentage of metabolic reads within each cell (Supplementary Fig. S3). Remarkably, metabolic gene expression patterns varied significantly across cell types, with malignant and myeloid cells displayed a higher percentage of metabolic reads compared to T and B cells, consistently observed across different cancer types. Unsupervised clustering based on metabolic genes effectively distinguished main cell lineages, resembling the results based on highly variable genes (Fig. 1d). Integrating cells across all 10 datasets using metabolic genes resulted in cells of the same major lineage being correctly grouped together, regardless of their dataset origin (Supplementary Fig. S4 and b). Furthermore, metabolic features predict cell identities independently of cell lineage markers, as indicated by high area under the receiver operating characteristic curve (AUC) values (Supplementary Fig. S4c). Additionally, metabolic pathway and regulon scores could also distinguish cell lineages, albeit to a lesser extent (Fig. 1d). Together, these results collectively underscore the robustness of metabolic differences across the main cell lineages.

Another insight from clustering analysis revealed that malignant cells formed sample-specific clusters, while non-malignant cells formed cell type-specific clusters, suggesting higher intertumour metabolic heterogeneity of malignant cells (Supplementary Fig. S5a–c). To validate this finding, we utilized a principal components (PC)-based correlation coefficient to quantify cell-to-cell metabolic similarity (Methods for details). Using this metric, we observed that malignant cells exhibited lower intertumour metabolic similarity than intratumour metabolic similarity, a trend not significant for non-malignant cells (Supplementary Figs. S5d and S6). This was further corroborated by ROGUE^35^ analysis, assessing the purity of single-cell populations (Supplementary Fig. S5e). Furthermore, cells from patients within the same cancer subtypes exhibited higher metabolic similarity than those from different subtypes (Supplementary Fig. S5f), highlighting the multilayered nature of tumour metabolic heterogeneity.

### Cell type-specific metabolic properties in TME

**Fig. 2:**
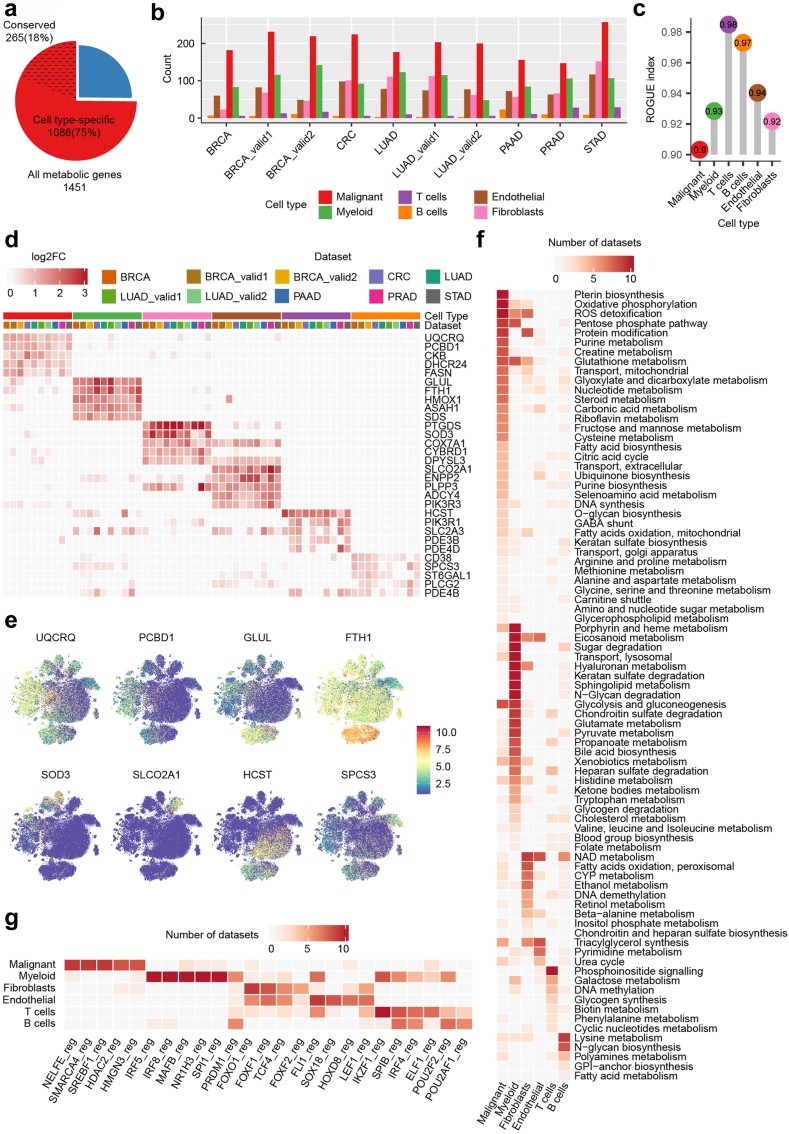
Cell type-specific metabolic properties in TME. Cell type-specific metabolic properties were identified using Wilcoxon rank sum test, see Methods for details. **(a)** Pie chart showing the proportion of cell type-specific metabolic genes in all metabolic genes. **(b)** Bar plot showing the number of cell type-specific metabolic genes for each cell type in each dataset. **(c)** Lollipop plot showing the similarity of metabolic gene profiles among cancer types for each major cell lineage by ROGUE. **(d)** Heatmap showing the conserved metabolic signature genes for distinct cell types, color indicating log_2_ fold change compared with all other cells. **(e)** t-SNE plots for combined datasets based on metabolic genes as in Supplementary Fig. S4a, color-coded according to expression levels of representative metabolic markers of main cell lineages. **(f)** Heatmap showing conserved metabolic signature pathways, color indicating the number of datasets in which a pathway is significantly upregulated in specific cell type. **(g)** Heatmap showing the top conserved metabolic signature regulons for each cell type.

Given the substantial metabolic variations among cell types, our focus shifted to unravelling cell type-specific metabolic properties within tumour tissues (Supplementary Table S4). A staggering 75% of all metabolic genes exhibited differential expression levels across cell types in at least one dataset, with 18% demonstrating consistency across all tumours (Fig. 2a). Malignant cells showcased the highest number of highly expressed metabolic genes, while T and B cells exhibited relatively few metabolic gene signatures (Fig. 2b). ROGUE analysis highlighted the pronounced metabolic heterogeneity of malignant cells across datasets, contrasting with the higher homogeneity observed in T and B cells (Fig. 2c). Specific metabolic genes, such as *UQCRQ*/*PCBD1*, *GLUL*/*FTH1*, *SOD3*, *SLCO2A1*, *HCST* and *SPCS3*, consistently exhibited high expression in malignant, myeloid, fibroblast, endothelial, T and B cells, respectively (Fig. 2d and e). Notably, some of these genes, including *FTH1* and *SLCO2A1*, play pivotal roles in tumour progression.^36^^,^^37^ Nevertheless, further investigations are warranted to elucidate the functions of most cell type-specific metabolic genes in the TME. Certain metabolic genes exhibited substantial heterogeneity across cancer types (Supplementary Fig. S7). For example, *SOD3*, specific to fibroblasts in most tumours, is expressed at high levels in normal epithelial cells and stellate cells but suppressed in malignant cells of PAAD, associated with the promotion of an aggressive phenotype.^38^ *LDHB*, widely expressed across different cell types, appears to be suppressed in malignant cells of BRCA and PAAD. To validate our scRNA-seq findings, we reanalysed published bulk RNA-seq data and mass cytometry data. Rohatgi et al.^39^ identified cancer- and stroma-specific metabolic genes in bulk tumours samples using a deconvolution approach. We confirmed the cell type specificity of these genes within our datasets (Supplementary Fig. S8a), and provided a more nuanced resolution for stroma-specific metabolic genes, which were predominantly expressed in myeloid cells. Additionally, Hartmann et al. identified cell type-specific proteins using mass cytometry data from CRC samples.^7^ We found consistent metabolic markers of malignant cells in both their data and ours (Supplementary Fig. S8b–e), demonstrating the robustness of these markers at both the gene and protein levels.

To provide a systematic view of the metabolic properties of each cell type, we identified cell type-specific metabolic pathways. Of the 96 metabolic pathways investigated, 83 exhibited significant differences across cell types (Fig. 2f), reflecting divergence in almost all aspects of their metabolic networks. Malignant cells displayed the largest number of upregulated metabolic pathways compared to other cell types, consistent with the gene-level results. Key pathways, including pterin biosynthesis, oxidative phosphorylation (OXPHOS), ROS detoxification, pentose phosphate pathway (PPP), purine metabolism, glycolysis and gluconeogenesis were identified as specific pathways of malignant cells in at least 80% (8/10) of datasets, aligning with their rapid proliferation and high energy demands. Despite increased glutaminolysis being reported as a hallmark of cancer metabolic reprogramming,^3^ our finding revealed that the glutamate metabolism pathway exhibited the highest activity in myeloid cells rather than malignant cells in most datasets (Supplementary Fig. S9). Further investigation of core enzyme genes indicated limited expression of *GLS* and *GLUD1* across all cell types, while *GLUL* consistently exhibited high expression levels in myeloid cells, signifying active glutamine anabolism triggered by excessive glutamine uptake by malignant cells.^40^

To explore cell type-specific regulation of metabolic networks, we reconstructed and identified cell type-specific metabolic regulons in each dataset independently (Fig. 2g). Among them, NELFE and SMARCA4, known participants in the progression of multiple cancers,^41^^,^^42^ raised questions about their specific role in metabolic regulation. Similarly, IRF5, implicated in the metabolic response of airway macrophage,^43^ hinted at potential functions in cancer, demanding further validation.

### Cell type-specific metabolic reprogramming patterns in TME

**Fig. 3:**
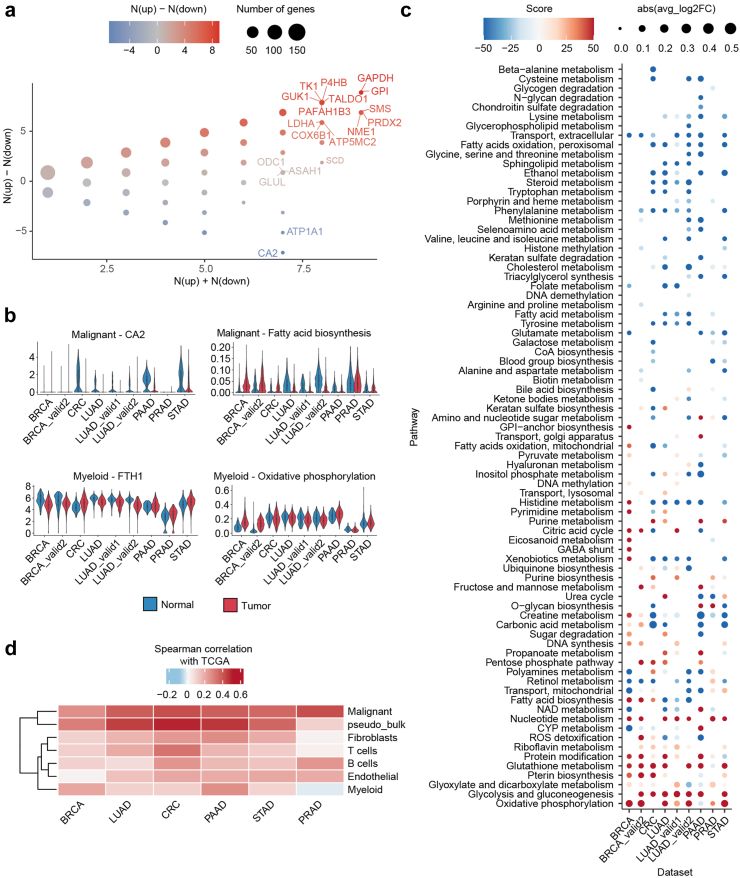
Cell type-specific metabolic reprogramming patterns. Cell type-specific metabolic reprogramming patterns were identified using Wilcoxon rank sum test, see Methods for details. **(a)** Dot plot showing the alteration of metabolic gene expression in malignant cells compared to epithelial cells in normal tissues. N (up) indicates the number of datasets in which a gene is significantly upregulated, and N (down) indicates the number of datasets in which a gene is significantly downregulated. The dot is coloured according to N (up) – N (down), and the dot size indicates the number of overlapping genes. **(b)** Violin and box plots showing the levels of representative genes and pathways between tumours and normal tissues across cancer types. **(c)** Dot plot showing the alteration of metabolic pathways in malignant cells compared to epithelial cells in normal tissues. The dot is coloured according to differential score, and the dot size indicates the absolute value of log_2_ fold change. The absolute value of differential score equals to -log_10_(FDR), and a positive value indicates upregulated, while a negative value indicates downregulated. **(d)** Clustered heatmap showing the correlation between cellular level and individual-level metabolic reprogramming, which is defined as the Spearman correlation coefficient between two groups of log_2_ fold change values.

Incorporating normal tissues allowed us to investigate cell type-specific metabolic reprogramming patterns across cancers (Supplementary Table S5), referring to the metabolic alterations compared to corresponding cell types in normal tissues. For malignant cells, consistently upregulated metabolic genes were predominantly associated with cancer hallmark pathways, including glycolysis (*GAPDH*, *GPI* and *LDHA*), OXPHOS (*COX6B1* and *ATP5MC2*), and nucleotide metabolism (*TK1*, *GUK1*, *NME1*) (Fig. 3a). The most representative downregulated gene, *CA2* (Fig. 3b), encodes one of several isozymes of carbonic anhydrase. While *CA9*, a family member, is upregulated by hypoxia in many cancers, and its inhibitors are used as anticancer drugs.^44^ *CA2* displayed an opposite expression trend to *CA9*, warranting further investigation of its role in cancer. Additionally, several genes exhibited significant expression differences across cancers, such as *SCD* and *ASAH1*, downregulated in LUAD but upregulated in other cancers. Pathway-level results corroborated the gene-level findings (Fig. 3c). Despite the common notion of malignant cells favouring a switch from OXPHOS to glycolysis, our findings revealed a significant upregulation of OXPHOS in most tumours compared to normal tissues, highlighting the metabolic plasticity of malignant cells. Pathways associated with lipid and amino acid metabolism were generally downregulated across cancers, particularly in LUAD, indicating inherent characteristics of this cancer type.^16^ Conversely, fatty acid biosynthesis and the citric acid cycle (CAC) displayed heterogeneity across cancer types (Fig. 3b and c), reflecting the tissue-specific biology of their tissue of origin.

Metabolic reprogramming patterns of non-malignant cells yielded unexpected results. The glycolysis pathway and associated genes were upregulated in nearly all cell types within tumours compared to their counterparts in normal tissues (Supplementary Fig. S10 and S11). This suggests an overall increase in the energy demand of the TME. Notably, as a metabolic feature of myeloid cells, *FTH1* was upregulated in the CRC and STAD datasets but downregulated in all BRCA and LUAD datasets compared to corresponding normal tissues (Fig. 3b). Many metabolic pathways of myeloid cells exhibited opposite regulation in BRCA and LUAD, including OXPHOS, hinting at potential differences in the functional phenotype of myeloid cells in these two cancer types.

To explore the alteration in degree of metabolic heterogeneity between tumours and normal tissues, we assessed the cell-to-cell metabolic similarity in these two groups. However, no significant differences were observed for most cell types (Supplementary Fig. S12a and b). Since metabolic reprogramming patterns at the individual level has been extensively explored,^9^, ^10^, ^11^ we investigate the correlation of results obtained at cellular level with those from individual level. Individual-level results were first reproduced using TCGA-GTEx data (Supplementary Fig. S12c), and then their correlations with cellular-level results were determined (Fig. 3d). We found that the metabolic reprogramming of each cell type was indeed quite different from individual-level results (maximum correlation coefficient <0.5), indicating that the individual-level studies masked the differences among diverse cell types. Pooled pseudo-bulk samples showed the highest similarity with real bulk samples, followed by malignant cells, suggesting that malignant cells may dominate the shaping of the tumour metabolic microenvironment.

### Intertumour metabolic heterogeneity of malignant cells and associated factors

Given the higher metabolic heterogeneity of malignant cells compared to non-malignant cell types, we conducted an in-depth exploration of the pathways involved and factors associated with them. Examining the correlation between averaged pathway scores and individual clinical metrics revealed no tight association with age and sex. However, fatty acid metabolism appeared to be linked to cancer stage in several datasets (Supplementary Fig. S13a). Pterin biosynthesis and DNA synthesis exhibited correlation with the CNV score, indicating heterogeneity in chromosomal aberrations among different patients.

Another significant factor associated with metabolic heterogeneity is the cancer subtype from which the tumour cells originated. For instance, malignant cells from ERBC exhibited higher levels of the carnitine shuttle pathway and lower levels of other pathways, including triacylglycerol synthesis, pyrimidine metabolism, and glycolysis, compared to TNBC (Supplementary Fig. S13b and c). These differences were supported by RNA-seq and metabolomics data from cultured cancer cell lines in the CCLE database (Supplementary Fig. S13d and e). Malignant cells from mismatch repair-deficient (MMRd) and mismatch repair-proficient (MMRp) CRC also demonstrated significant differences in multiple pathways (Supplementary Fig. S13f), potentially linked to disparities in their mutational burden.

### Intratumour metabolic heterogeneity of malignant cells revealed by metabolic meta-programs

**Fig. 4:**
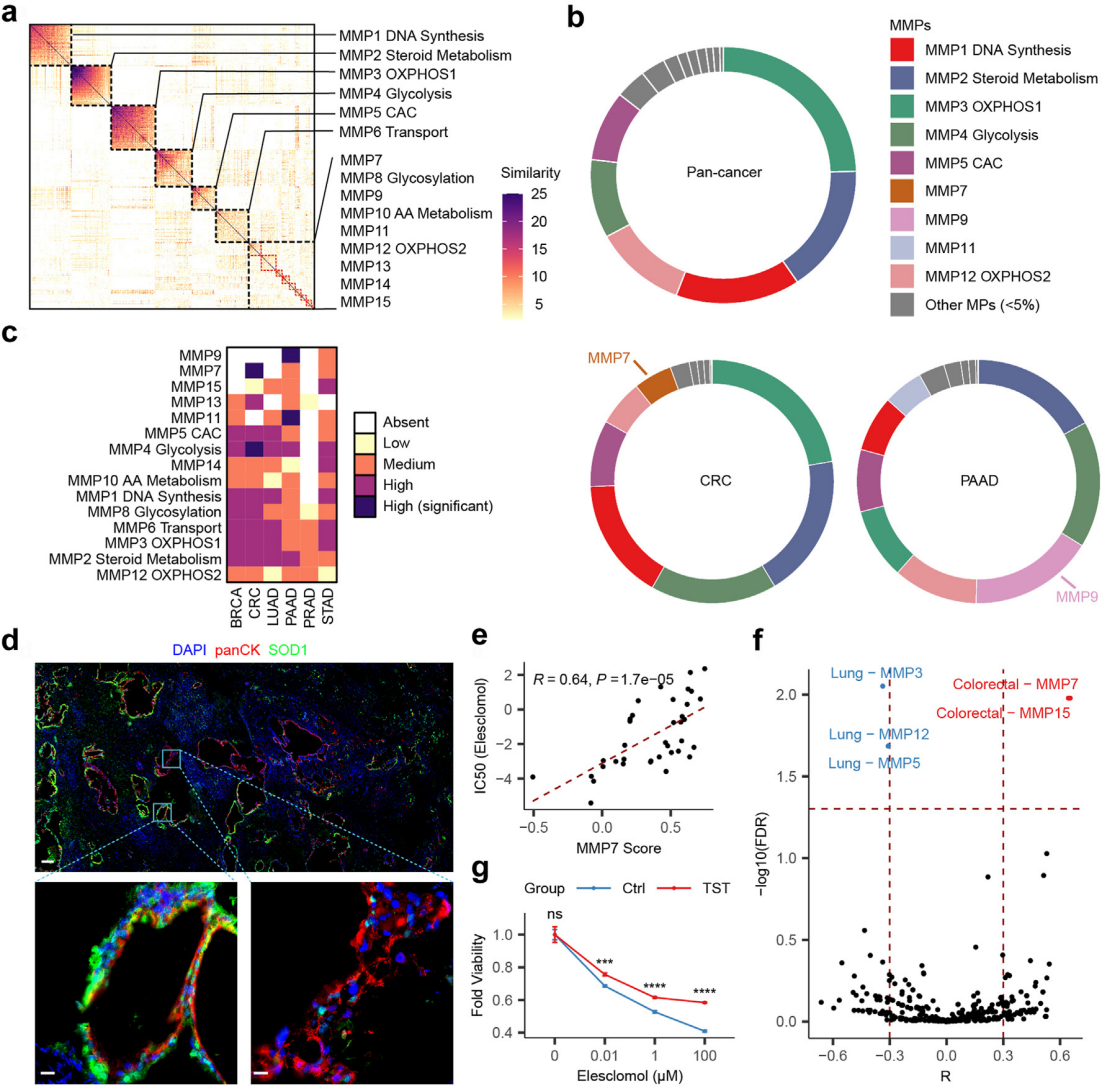
MMPs for malignant cells. **(a)** Heatmap showing Jaccard similarity indices for comparisons among robust NMF programs, which were ordered by clustering and grouped into MMPs. Only clustered NMF programs were shown. **(b)** The circles reflect the proportion of cells assigned to each MMP in all tumours (top), CRC (left bottom) and PAAD (right bottom). The cancer-specific MMPs for CRC and PAAD were labelled. **(c)** Abundance of each MMP (rows) in each cancer type (columns), defined as absent, low, medium, high or high and significant. **(d)** Validation of representative gene (*SOD1*) expression of MMP9 by IHC in PAAD. Scale bars, 200 μm (top) and 20 μm (bottom). The ratio of fluorescence intensity of SOD1/panCK, quantified using ImageJ, is 0.73 (bottom left) versus 0.11 (bottom right). **(e)** Scatter plot shows the correlation between MMP7 scores and IC50 values of elesclomol in colorectal cancer cell lines in CCLE datasets. Pearson correlation coefficients and *P* values from linear models are shown. **(f)** Volcano plot of MMPs showing significant correlations (Pearson correlation coefficients >0.3 or < −0.3, and FDR <0.05) with IC50 values of elesclomol in all cancer cell lines in CCLE datasets. Significant correlations are labelled in the plot. **(g)** The effect of TST overexpression on drug response to elesclomol at 24 h in HCT116 cells. Data are represented as mean ± SEM. Compared to negative control (Student’s t test), ns means not significant; ∗∗∗*P* < 0.001; ∗∗∗∗*P* < 0.0001.

The functional diversity of tumour cells within the local microenvironment prompted an investigation into intratumour metabolic heterogeneity. To accomplish this, we defined MMPs using an algorithm described by Gavish et al.^27^ with minor modifications. Briefly, NMF was utilized to characterize metabolic gene expression programs that vary within each tumour, and then robust NMF programs shared by tumours were clustered into MMPs. This resulted in 15 MMPs (Fig. 4a and Supplementary Table S6), each summarized by its top 30 genes that are coordinately upregulated in subpopulations of cells within many tumours. Several MMPs resembled the meta-programs identified by Gavish et al. but emphasized the intratumour heterogeneity of metabolic processes (Supplementary Fig. S14a), while others are newly identified. The most frequently identified MMP was annotated to DNA synthesis, consistent with the proliferative nature of tumours. Other broadly identified MMPs were annotated to steroid metabolism, OXPHOS, glycolysis, CAC and transport, all of which were fundamental cellular metabolic processes. Several less frequent MMPs could not be annotated to specific pathways, underscored the intricate interplay between metabolic processes.

To explore MMP significance, we investigated their associations with cancer hallmarks (Supplementary Fig. S14b). MMPs related to DNA synthesis, OXPHOS, and CAC exhibited positive correlations with DNA repair but negative correlations with angiogenesis, apoptosis, epithelial–mesenchymal transition (EMT), and inflammatory response, highlighting their pivotal roles in tumour progression. Examining clinical relevance, MMPs associated with overall survival were identified (Supplementary Fig. S14c). Apart from glycolysis related MMP4 and DNA synthesis related MMP1, an unannotated MMP11 also associated with worse overall survival, emphasizing the importance of related metabolic processes.

Although MMPs were defined as clusters of recurrent NMF programs in multiple tumours, they were not needed to be shared across cancer types. Thus, we investigated the abundance of each MMP in each cancer type (Fig. 4b and c, Supplementary Table S7). We found that a great part of MMPs were indeed identified in most cancer types, indicating that intratumour metabolic heterogeneity shows recurrent patterns across cancer types. Nonetheless, certain MMPs exhibited significant context specificity, not necessarily associated with the specificity of high expression (Supplementary Fig. S15). For instance, MMP9, significantly enriched in PAAD (Fig. 4c and Supplementary Fig. S16), demonstrated intratumour heterogeneity confirmed by immunohistochemistry (IHC) (Fig. 4d). Further examination of MMP9 via spatial transcriptomics (ST) data from our lab (unpublished) and a previous study^34^ (Supplementary Fig. S17) revealed that it is specifically enriched in pancreatic intraepithelial neoplasia (PanIN) lesions, which is a histologically well-defined precursor to PAAD, hinting at a potential benign metabolic marker for PAAD. As a second example, MMP7 is enriched in CRC (Fig. 4c and Supplementary Fig. S16) and exhibited medium abundance in other digestive system neoplasms. To explore the clinical significance of these cancer type-specific MMPs, we systematically investigated their correlation with drug sensitivity using CCLE datasets (Supplementary Fig. S18a). While most significant correlations were observed in lung cancer cell lines, potentially due to the larger sample size, unexpected correlations were found, such as potential resistance to elesclomol in colorectal cancer cell lines with higher MMP7 expression (Fig. 4e). Elesclomol is a copper ionophore and reported to induce a new form of programmed cell death, called cuproptosis, via a mitochondrial respiration dependent way. Interestingly, our analysis also found that lung cancer cell lines with higher OXPHOS (MMP3 and MMP12) and CAC (MMP5) were more sensitive to elesclomol (Fig. 4f), consistent with previous study,^45^ supporting the reliance of our analysis results. Thus, we sought to further validate the specific correlation between MMP7 and elesclomol in colorectal cancer. We found that HCT116 cells with overexpressed MMP7 representative genes (*TST* and *FUT3*) were indeed more resistant to elesclomol treatment compared to control (Fig. 4g and Supplementary Fig. S18b), implying that patients with CRC with who have lower MMP7 scores may be more likely to benefit from elesclomol treatment. However, further investigation is required to elucidate the mechanism by which these genes are involved in cuproptosis.

### Master regulators of malignant cell MMPs

Given the tight clinical associations observed for MMPs of malignant cells, we sought to explore the regulation of these MMPs. Through an examination of the correlation between regulon scores and MMP scores within each tumour, we identified highly recurrent master regulators across caner types (Supplementary Fig. S19). This analysis not only revealed expected regulators for annotated MMPs, such as the MYB family transcription factor (MYBL2) for DNA synthesis and the sterol regulatory element-binding transcription factor (SREBF2) for steroid metabolism but also uncovered many putative new regulatory interactions.

**Fig. 5:**
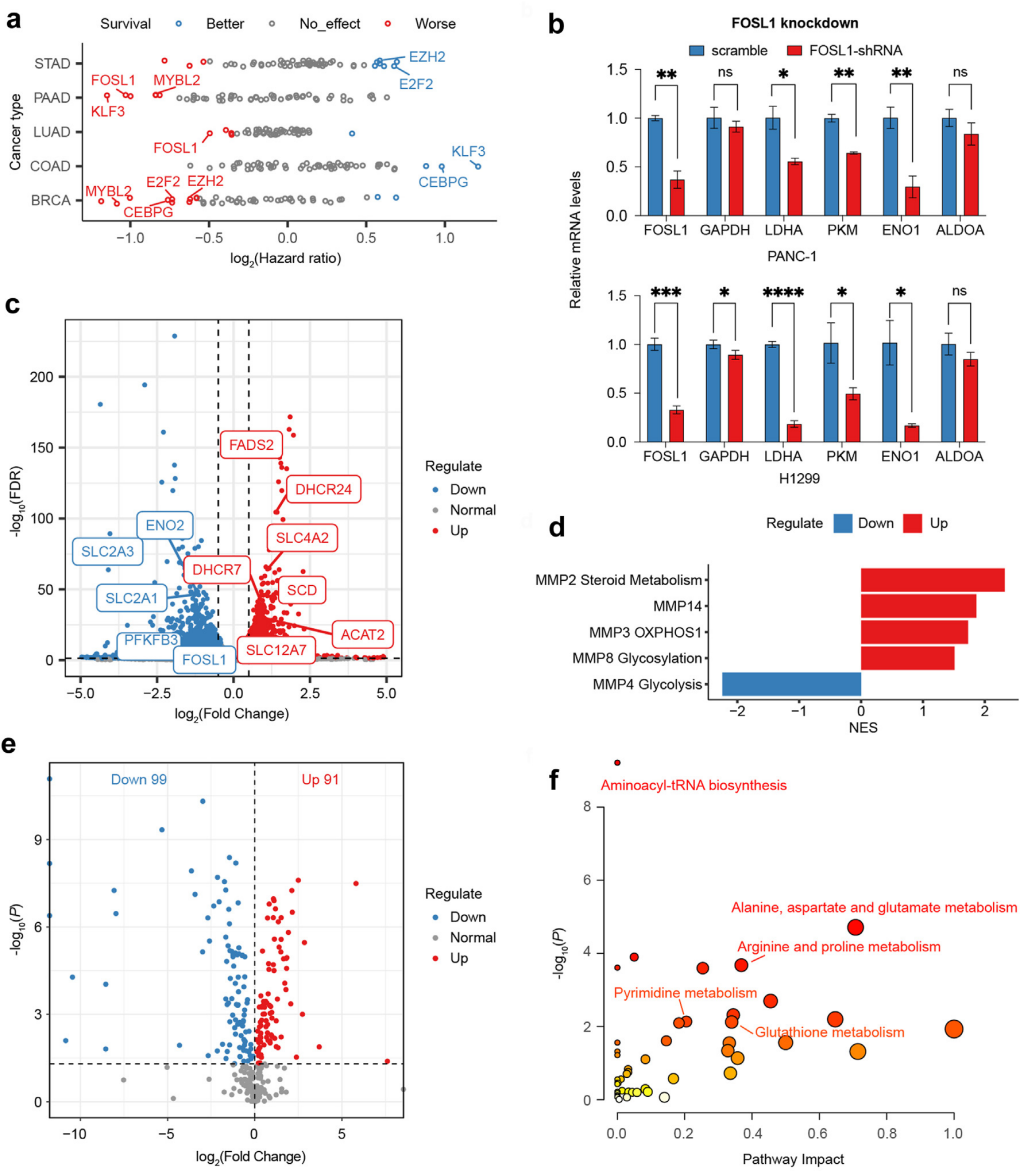
Validation of master regulators of malignant cell metabolism. **(a)** Clinical associations of the expression of identified regulators with patient overall survival in TCGA. Significant associations (*P* value < 0.05) are coloured with red or blue. Red indicates that higher expression of a regulator is associated with worse prognosis and blue indicates the opposite. The hazard ratios and *P* values were calculated using Cox regression models with the age, sex and clinical stages corrected. **(b)** Bar plot showing the change in relative mRNA levels of *FOSL1* and glycolytic enzyme genes between knockdown cell lines and controls. The experiment was performed in triplicate, and the data are shown as the mean ± SEM. Unpaired two-tailed Student’s t test was employed to calculated the statistical significance. ns, not significant; ∗*P* < 0.05; ∗∗*P* < 0.01; ∗∗∗*P* < 0.001; ∗∗∗∗*P* < 0.0001. **(c)** Volcano plot of genes showing significant changes (FDR <0.05 and |log_2_FC| > 0.5) after *FOSL1* knockdown in PANC-1 cell lines. The x-axis shows log_2_ fold change (log_2_ FC), and the y-axis shows -log_10_(FDR). Representative metabolic genes are labelled in the plot. **(d)** Bar plot showing significant MMPs (*P* < 0.05) identified by GSEA. The x-axis represents the normalized enrichment score (NES). **(e)** Volcano plot of metabolites showing significant changes (Student’s t test, *P* < 0.05) after *FOSL1* knockdown in PANC-1 cell lines. **(f)** Metabolic pathways showing significant changes in *FOSL1* knockdown cell lines. The dot color indicates the level of significance, and the dot size indicates the pathway impact. The *P* value was calculated from the enrichment analysis in MetaboAnalyst.

To screen regulators with clinical significance for experimental verification, we assessed the association of the expression of these regulators with overall survival (Fig. 5a). Remarkably, FOSL1 (in PAAD and LUAD) and MYBL2 (in PAAD and BRCA) consistently showed associations with worse survival across multiple cancer types. *FOSL1* was inferred as a potential regulator of MMP4 (Glycolysis), MMP6 (Transport), MMP10 (Amino acid metabolism) and MMP11. To experimentally validate these findings, we initiated shRNA knockdown experiments targeting *FOSL1* in the LUAD cell line H1299 and the PAAD cell line PANC-1 (Fig. 5b and Supplementary Table S8). The results demonstrated a significant reduction in the expression of glycolytic enzymes upon *FOSL1* knockdown. Subsequent RNA-seq analysis of the *FOSL1* knockdown PANC-1 cell line revealed downregulation of glycolytic genes (*ENO2* and *PFKFB3*) and transporters (*SLC2A1* and SLC2A3), accompanied by upregulation of genes associated with fatty acid and steroid metabolism (*FADS2*, *DHCR24* and *SCD*) (Fig. 5c). Gene set enrichment analysis^46^ (GSEA) further supported the dysregulation of these MMPs (Fig. 5d). Untargeted metabolomics provided insights into the metabolic changes induced by *FOSL1* knockdown, identifying metabolites with significant differences compared to scrambled shRNA controls (Fig. 5e and Supplementary Table S9). Pathway-based enrichment analysis of these differential metabolites suggested a metabolic disorder involving multiple amino acids indicated by MMP10 (Fig. 5f). While *FOSL1* has been reported as an oncogene in multiple cancers including PAAD,^47^ its role in metabolic regulation remains unclear. Further studies will be essential to determine whether its association with prognosis is dependent on its regulation of tumour metabolism.

### Metabolic heterogeneity of non-malignant cells in TME

**Fig. 6:**
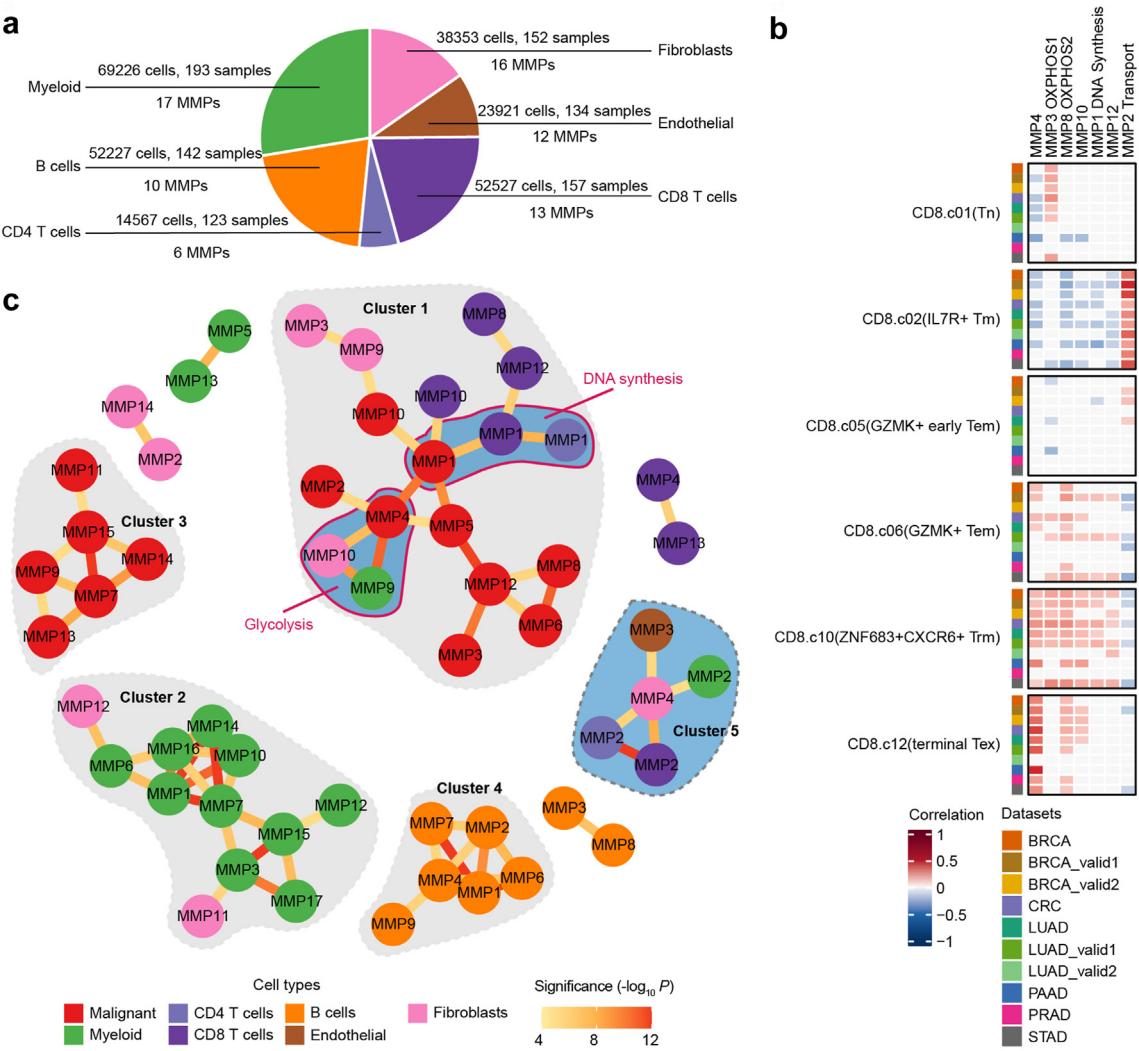
MMPs of non-malignant cells and metabolic crosstalk in TME. **(a)** Pie chart depicting the cell proportion of main non-malignant cell lineages in all tumours, with the number of cells, tumour samples and MMPs labelled. **(b)** Heatmap showing the correlations between MMP scores and signature scores of CD8+ T cell metaclusters during exhaustion paths. Pearson correlation coefficients were calculated within each tumour and then averaged across tumours within each dataset. Significant correlations (Mean Pearson correlation coefficient >0.1 or < −0.1, and FDR <0.05) are coloured according to the correlation coefficients. Only MMPs with consistently significant correlations with at least one signature in more than 40% (4/10) datasets were shown. **(c)** Network visualization of positive correlations (edges) between MMPs (nodes) from different cell types. Node color indicates cell types and edge color indicates the correlations’ significance.

Shifting our focus to the intratumour metabolic heterogeneity of non-malignant cells, we defined MMPs in various common cell types within the TME (Fig. 6a and Supplementary Table S6). MMPs of non-malignant cell types exhibited limited similarity to those of malignant cells, with exceptions in several DNA synthesis and OXPHOS related MMPs (Supplementary Fig. S20), emphasizing the diverse metabolic properties in different cell types within the TME. Context specificity analysis revealed that most non-malignant MMPs were shared across cancer types (Supplementary Fig. S21), underscoring the inherent metabolic heterogeneity of these cells.

To understand the significance of non-malignant MMPs, we explored their association with immune cell differentiation. Particularly, we focused on tumour-infiltrating CD8^+^ T cells, known to undergo T cell exhaustion, a state that compromises the efficacy of immunotherapies. Zheng et al.^22^ inferred two paths of T cell exhaustion via pan-cancer analysis: the first path (P1) through *GZMK*^+^ effector memory T (T_em_) cells and the second path (P2) through *ZNF683*^+^*CXCR6*^+^ tissue-resident memory T (T_rm_) cells (Supplementary Fig. S22a). Signature genes of CD8^+^ T cell metaclusters during exhaustion paths exhibited a gradual transition of gene expression patterns during T cell exhaustion (Supplementary Fig. S22b). Correlating these signature scores with MMP scores (Fig. 6b) also revealed a gradual transition of metabolic patterns. Naïve T cells exhibited high OXPHOS activity, *IL7R*^+^ memory T cells (T_m_) were characterized by MMP2, and terminal exhausted T cells (T_ex_) signatures correlated with MMP4, involving genes related to glycolysis, folate, and fatty acid metabolism.

For CD4^+^ T cells, naïve T (T_n_) cells differentiated into *IFNG*^+^ follicular helper T cell (T_fh_)/T helper 1 (T_h_1) dual-functional cells, *TNFRSF9*^+^ regulatory T (T_reg_) cells, and terminally differentiated effector memory or effector (T_emra_) cells (Supplementary Fig. S22c). Notably, *TNFRSF9*^+^ T_reg_ cells were significantly characterized by MMP3 expression, which included various pathways like sulphur compound metabolic process (Supplementary Fig. S22d). Exploring the link between individual metabolic genes and the signature scores of CD4^+^ T cell metaclusters revealed significant differences in T_reg_ scores between cells grouped by the expression of key components of MMP3, including *ENTPD1*, *GLRX*, *GCNT1* and *HACD1* (Supplementary Fig. S22e), suggesting the involvement of these metabolic genes in the acquisition of immunosuppressive properties by CD4^+^ T cells.

In vitro, macrophages exhibit M1/M2 dualistic polarization states with co-expression of M1 and M2 signature genes observed in tumour-associated macrophages (TAMs).^48^^,^^49^ M1 and M2 scores, however, displayed different correlation patterns with metabolic processes (Supplementary Fig. S22f). M2 scores were positively correlated with multiple MMPs, including mixed MMP1 and CAC related MMP7, while being negatively correlated with DNA synthesis related MMP3. In contrast, M1 scores exhibited fewer significant associations. Beyond M1/M2 phenotypes, Zhang et al.^24^ defined dichotomous functional phenotypes (angiogenesis/phagocytosis) of TAMs in CRC. Interestingly, angiogenesis and phagocytic signatures showed similar correlation patterns as M1 and M2 signatures, respectively, with the exception of glycolysis associated MMP9, which positively correlated with angiogenic and M2 signatures, aligning with their association with poor prognosis.^49^ Significant differences in phagocytic signature scores were identified between cells grouped by the expression of key components of MMP1, such as *HMOX1*, *ASAH1* and *SDS* (Supplementary Fig. S22g). These genes, as well as GLUL, were previously identified as significant metabolic signatures of myeloid cells (Fig. 2d), suggesting a potential role in the protumour function of TAMs.^50^

B cells also play crucial roles in adaptive immune system, primarily through antibody generation. Traditionally, antibody-secreting cells (ASCs) were thought to originate from germinal centre response. However, Ma et al. identified an alternative extra-follicular differentiation pathway in a pan-cancer analysis of tumour-infiltrating B cells, leading to atypical memory (AtM) B cells, which display an exhausted and bystander phenotype within TME.^23^ Our analysis of B cell subclusters revealed that geminal centre B (GCB) cells, known for their rapid proliferation, exhibit active DNA synthesis (Supplementary Fig. S22h). In contrast, AtM B cells showed metabolic patterns similar to those of naïve B cells, suggesting a dysfunction state. Unlike Ma et al.’s findings, we did not observe a strong cancer type preference for these metabolic patterns, nor did we identify a significant association between glutamine metabolism and AtM B cells, possibly due to the limited number of cancer types analysed.

### Metabolic associations between cell types in TME

In the intricate landscape of the TME, diverse cell types may engage in metabolic interactions, competing for nutrients or providing each other with metabolic substrates. To unravel these effects, we explored the co-occurrence of MMPs of different cell types, determined by the correlation of centred fractions of cells with high scores for each MMP in each tumour (Methods). The positive correlation network revealed five clusters (Fig. 6c). The largest cluster (Cluster 1) comprised MMPs from various cell types, with malignant cells acting as the hub, indicating extensive crosstalk between tumour cells and non-malignant cells in the TME. Within this cluster, two subclusters corresponding to specific metabolic processes were also highlighted, one associated with DNA synthesis in malignant cells and two subtypes of T cells, while the other related to glycolysis in malignant cells, fibroblasts and myeloid cells. These correlations suggest a potential synergistic effect among these cell types. The smallest clusters (Cluster 5) consisted of five unannotated MMPs, each originating from different cell types. To uncover the potential functions of this cluster, we merged the genes of these MMPs and conducted functional enrichment analysis using Gene Ontology (C5.GOBP) and Hallmark (H) gene sets from MsigDB. Apart from multiple metabolic process, cluster 5 exhibited enrichment in mTOC1 and TNFα signalling pathways (Supplementary Table S10), suggesting intricate regulation across different cell types under the influence of common signalling pathways. The remaining three clusters primarily consisted of MMPs specific to particular cell type, highlighting the intricate interweaving of the metabolic network within these cells.

### Subtyping of patients based on metabolic properties of specific cell types

Finally, we sought to uncover the potential clinical implications of cellular-level metabolic heterogeneity. We reconstructed cell type specific pseudo-bulk samples by aggregating gene expression profiles from all single cells within a given cell type of a particular tumour. Metabolic pathways were then scored for these cell type specific pseudo-bulk samples using gene set variation analysis (GSVA). To capture the underlying structure, pathways were initially clustered into five highly correlated modules based on correlations across all cell type specific pseudo-bulk samples (Supplementary Fig. S23a–d). Subsequently, we clustered all cell type specific pseudo-bulk samples according to these metabolic modules, revealing distinct branches primarily segregated by cell type (Supplementary Fig. S23e). Importantly, cell type specific pseudo-bulk samples originating from different datasets of the same cancer type clustered together, underscoring the limited impact of batch effects on our results.

**Fig. 7:**
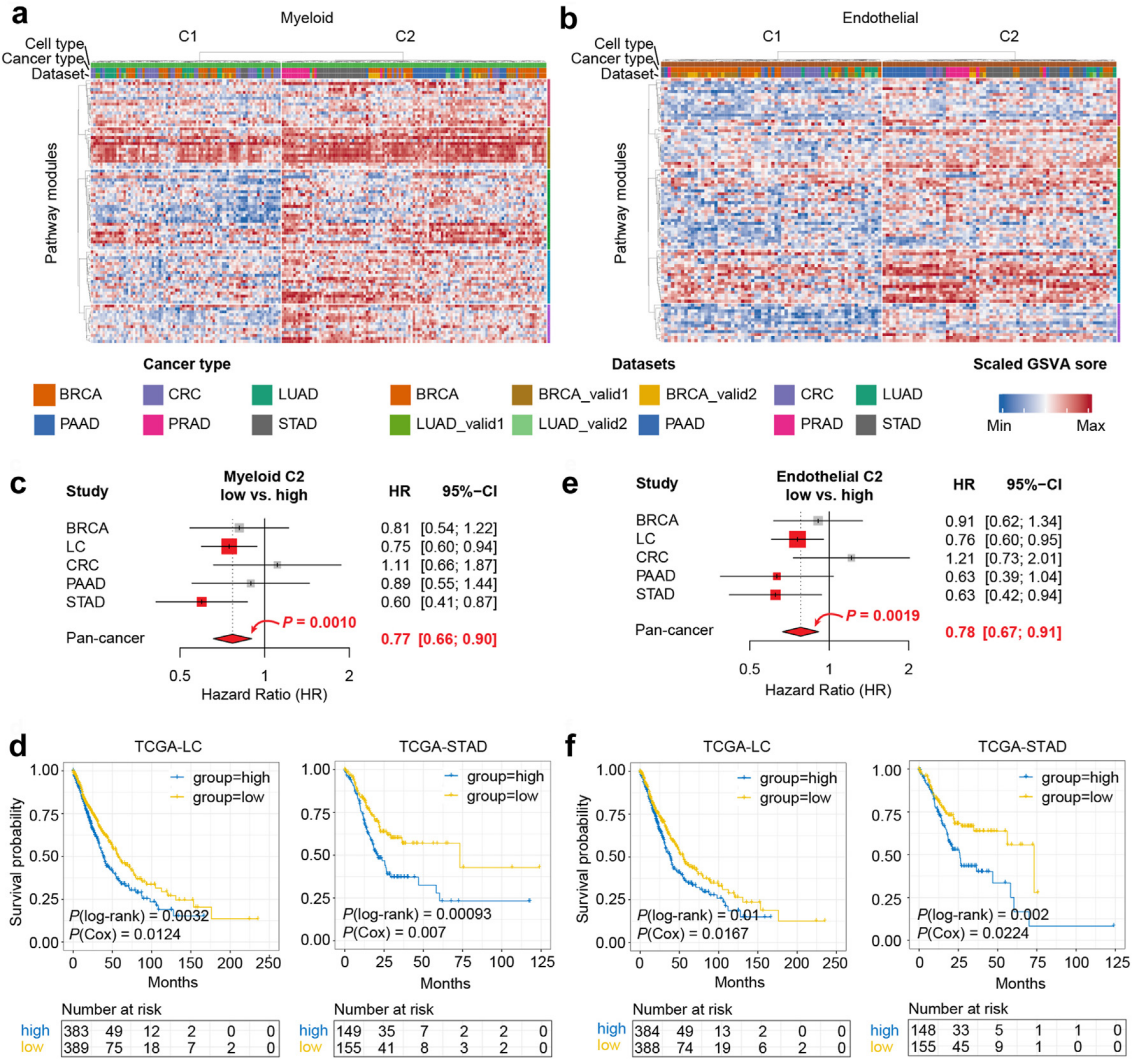
Metabolic subtypes of pan-cancer defined by cellular metabolic properties. **(a)** and **(b)** Heatmap showing the clustering results of pan-cancer samples based on the metabolic pathways of myeloid and endothelial cells. **(c)** Forest plot reporting the effect of myeloid C2 signatures on overall survival. The hazard ratios (HRs) and 95% confidence intervals (CIs) were calculated using Cox regression models with the age, sex and clinical stages corrected. The black solid line indicates hazard ratio 1 (meaning no effect). Red for *P* < 0.05. **(d)** Kaplan–Meier plots showing the survival curves of patients with LUAD and STAD grouped by levels of myeloid C2 signature scores. *P* values by both log-rank and Cox regression (with sex, age and clinical stage as covariates) are shown. **(e)** Similar plot as in **(c)** reporting the effect of endothelial C2 signatures. **(f)** Similar plot as in **(d)** for endothelial C2 signature scores.

We further refined the clustering by examining cell type specific pseudo-bulk samples for each cell type individually. For malignant specific pseudo-bulk samples, the clustering was dominated by tissue-of-origin. Specifically, C1 encompassed the majority of BRCA and LUAD samples, while samples from other tumours constituted C2 (Supplementary Fig. S24a). In contrast, non-malignant specific pseudo-bulk samples from various cancer types were intricately mixed (Fig. 7a and b; Supplementary Fig. S24b–d), with certain cancer types displaying discernible preferences. Notably, samples from C2 exhibited higher overall metabolic activities across all cell types compared to C1.

To elucidate the clinical significance of these metabolic types, we identified gene signatures corresponding to the aforementioned metabolic types. These signatures were then employed to stratify patients from The Cancer Genome Atlas (TCGA). Intriguingly, tumours characterized by higher myeloid and endothelial C2 signature scores exhibited worse overall survival across multiple cancer types, including lung cancer (LC), PAAD, and STAD, as well as in the pan-cancer model (Fig. 7c–f). This highlights the potential of these cellular-level metabolic types as a reference for understanding the metabolic heterogeneity within different cell types in the TME, offering valuable insights for patient stratification and treatment guidance.

## Discussion

Most tumours are complex ecosystems that evolve under diverse selective pressures, leading to the substantial spatiotemporal heterogeneity at genetic, epigenetic, transcriptional, phenotypic, and metabolic levels within the TME.^51^ Such heterogeneity promotes cancer progression and therapeutic resistance, yet offers potential targets for innovative treatments. Despite the well-established link between cell function and metabolic demands,^4^^,^^5^ comprehensive exploration of metabolic heterogeneity has been constrained by studies on bulk samples or limited by low throughput and sample size.^2^^,^^3^^,^^7^^,^^8^^,^^12^^,^^13^

Our study presents a single-cell level metabolic gene expression atlas, consolidating scRNA-seq data from nearly 300 tumour and normal samples. Although only six cancer types were included, they are among the most common with the highest morbidity and mortality. To ensure the reproducibility and credibility of our results, data from our lab and collaborators were combined with published datasets and uniform analysis pipelines were performed. As expected, consistent results were obtained from diverse datasets of the same cancer types.

Our pan-cancer analysis revealed a multilayered landscape of metabolic heterogeneity across cancers. The first layer unfolds across different cell types, unveiling unique metabolic properties and reprogramming patterns associated with specific functions. Our findings are consistent with previous studies but provide a more nuanced resolution. First, cancer cells exhibited the highest metabolic activity in many vital processes, including OXPHOS, compared to other cells within the TME.^14^^,^^39^ This highlights the metabolic plasticity of malignant cells, which can flexibly switch between OXPHOS and glycolysis to meet their metabolic demands. Second, non-malignant cells also exhibited Warburg-like phenotype compared to their counterparts in normal tissue, with myeloid cells displaying glycolytic activity comparable to that of malignant cells.^15^ This indicates a global elevation of energy demands within the TME, potentially leading to competition and cooperation for metabolic resources. Third, while most stroma-specific metabolic genes and pathways identified in previous studies^39^ showed specificity within certain stromal compartment, our analysis revealed that they were not uniformly expressed across all stroma cell types. However, some inconsistencies were also observed. For example, associated genes of metabolites in NAD^+^ biosynthesis pathway were reported to be broadly enriched in immune-related process.^52^ Nonetheless, in our analysis, the NAD metabolism pathway were predominantly activated in stromal cells (e.g., fibroblasts and endothelial cells, Fig. 2f), with activation in B cells observed only in certain datasets. These differences might stem from the varying methodologies, sample types, or the number of cancer types analysed, and they underscore the complexity of metabolic interactions within the TME.

The second layer delves into intratumour heterogeneity within specific cell types. By applying non-negative matrix factorization (NMF) to characterize robust metabolic gene expression programs and clustering them into metabolic meta-programs (MMPs), we found that most of them were shared across different cancers, indicating common metabolic states among cancer cell subtypes of different cancer. However, we also identified certain cancer type-specific MMPs. For example, MMP9 in PAAD was specifically enriched in premalignant lesions, indicating a potential benign metabolic marker. MMP7 in CRC was associated with sensitivity to elesclomol, offering insights into the mechanism study and clinical application of this promising anticancer drug.^45^^,^^53^ Additionally, our analysis of master regulators of cancer MMPs deepens our understanding of cancer metabolism regulation. Furthermore, investigation of MMPs in non-malignant cells reveals metabolic crosstalk under the influence of common signalling pathways.

The third layer of heterogeneity involves cell type-specific intertumour differences and their associations with clinical outcomes. Stratifying patients based on cell type-specific metabolic properties provide a more refined approach to patient segmentation compared to previous studies that relied on individual metabolic characteristics.^11^^,^^54^ This stratification could enhance the efficacy of existing treatments or lead to the development of new therapeutic modalities, by allowing for the targeting of specific metabolic processes in distinct cell types and cancer types. Such an approach holds promise for more personalized and effective cancer therapies, particularly in overcoming resistance mechanisms driven by metabolic heterogeneity.

Although our data provide a valuable resource for exploring the complexities of tumour metabolic heterogeneity, certain limitations remain. As mentioned earlier, the cancer types included in this study are not yet sufficient to depict a comprehensive picture of pan-cancer metabolic heterogeneity. Additionally, the complexity of data sources raises the possibility of selection bias in the scRNA-seq cell isolation protocols of certain datasets. For instance, we were unable to detect clear CNV signals in malignant cells from PRAD and STAD, and therefore adopted a compromised method for labelling malignant cells. Thus, more comprehensive datasets, collected and processed under standardized and uniform protocols, would further enhance the interpretability of these findings. Finally, the expression patterns of metabolic genes reflect only an indirect molecular layer of the metabolic network. Future studies incorporating additional data types, such as metabolomics and metabolic flux data in single-cell resolution, are anticipated to advance this field.

## Contributors

Y. Yin, Z.Z. and D.D. conceived the project and designed the major experiments. Z.Z. collected datasets and performed computational analysis. D.D. conducted the experiments. Y. Yuan, G.W. and J.L. performed metabolite profiling experiments. X.L. and L.C. carried out the IHC experiments and interpreted the data. Z.Z. and D.D. wrote the manuscript and Y. Yin thoroughly revised the manuscript. Y. Yin, Z.Z. and D.D. have directly accessed and verified the underlying data reported in the manuscript. Y. Yin was responsible for the decision to submit the manuscript. All authors read and approved the final manuscript.

## Data sharing statement

The availability of all data and codes used in this study were described in the method section “Data and code availability” and were all public available. The resources and tools used in our analyses were described in each method section.

## Declaration of interests

The authors declare no competing interests.
