# Supplementary material for: Single cell atlas reveals multilayered metabolic heterogeneity across tumour types
Source: eBioMedicine. 2024 Oct 10;109:105389. doi: 10.1016/j.ebiom.2024.105389 (PMC11663789; doi:10.1016/j.ebiom.2024.105389)
Supplement: Supplementary Figs. S1–S24 [file mmc1.pdf]

Single cell atlas reveals multilayered metabolic heterogeneity across tumour types

Zhe Zhou[1,#], Di Dong[1,#], Yuyao Yuan[1], Juan Luo[2], Xiao-Ding Liu[3], Long-Yun Chen[3], Guangxi Wang[1], and Yuxin Yin[1,*]

Table of content of supplementary material

Figure S1. Identification of main cell lineages.
Figure S2. Distinguish malignant cells from non-malignant epithelium.
Figure S3. Overall characteristics of metabolic gene expression.
Figure S4. Robust metabolic differences across major cell lineages.
Figure S5. Malignant cells show higher metabolic heterogeneity than non-malignant cells.
Figure S6. Quantification of cell-to-cell metabolic similarity in CRC dataset based on different methods.
Figure S7. Representative genes showing prominent heterogeneity across cancer types.
Figure S8. Validation of metabolic signature genes of malignant cells using mass cytometry data of CRC.
Figure S9. High level of glutamate metabolism in myeloid cells.
Figure S10. Gene signatures of metabolic reprogramming of non-malignant cells.
Figure S11. Pathway signatures of metabolic reprogramming of non-malignant cells.
Figure S12. Alteration of metabolic similarity between tumours and normal tissues.
Figure S13. Intertumour metabolic heterogeneity of malignant cells.
Figure S14. Associations between malignant MMPs and other factors.
Figure S15. Global (mean) expression vs. variability of MMP expression across different datasets.
Figure S16. MMP distribution in representative samples of PAAD and CRC.
Figure S17. MMP9 expression in ST data of PAAD.
Figure S18. MMPs and drug sensitivity.
Figure S19. MMP regulators for malignant cells.
Figure S20. Similarity between malignant MMPs and non-malignant MMPs.
Figure S21. Abundance of non-malignant MMPs.
Figure S22. MMP associations for immune cells.
Figure S23. Cluster of pathways and all cell type specific pseudo-bulk samples.
Figure S24. Metabolic subtypes of pan-cancer defined by cellular metabolic properties.

Table S1. Metadata for cohorts and samples, provided as an excel file.
Table S2. Canonical markers to assign the cell types, provided as an excel file.
Table S3. Curated metabolic genes and pathways, provided as an excel file.
Table S4. Cell type-specific metabolic properties, provided as an excel file.
Table S5. Cell type-specific metabolic reprogramming, provided as an excel file.
Table S6. MMPs identified in each cell type
Table S7. Number and percent of malignant cells assigned to each MMP, related to Figure 4b, provided as an excel file.
Table S8. shRNA and primer sequence used for knockdown experiments, provided as an excel file.
Table S9. Metabolites significantly changed in knockdown cell lines compared to controls, provided as an excel file.
Table S10. Enriched pathways for Cluster 5, provided as an excel file.

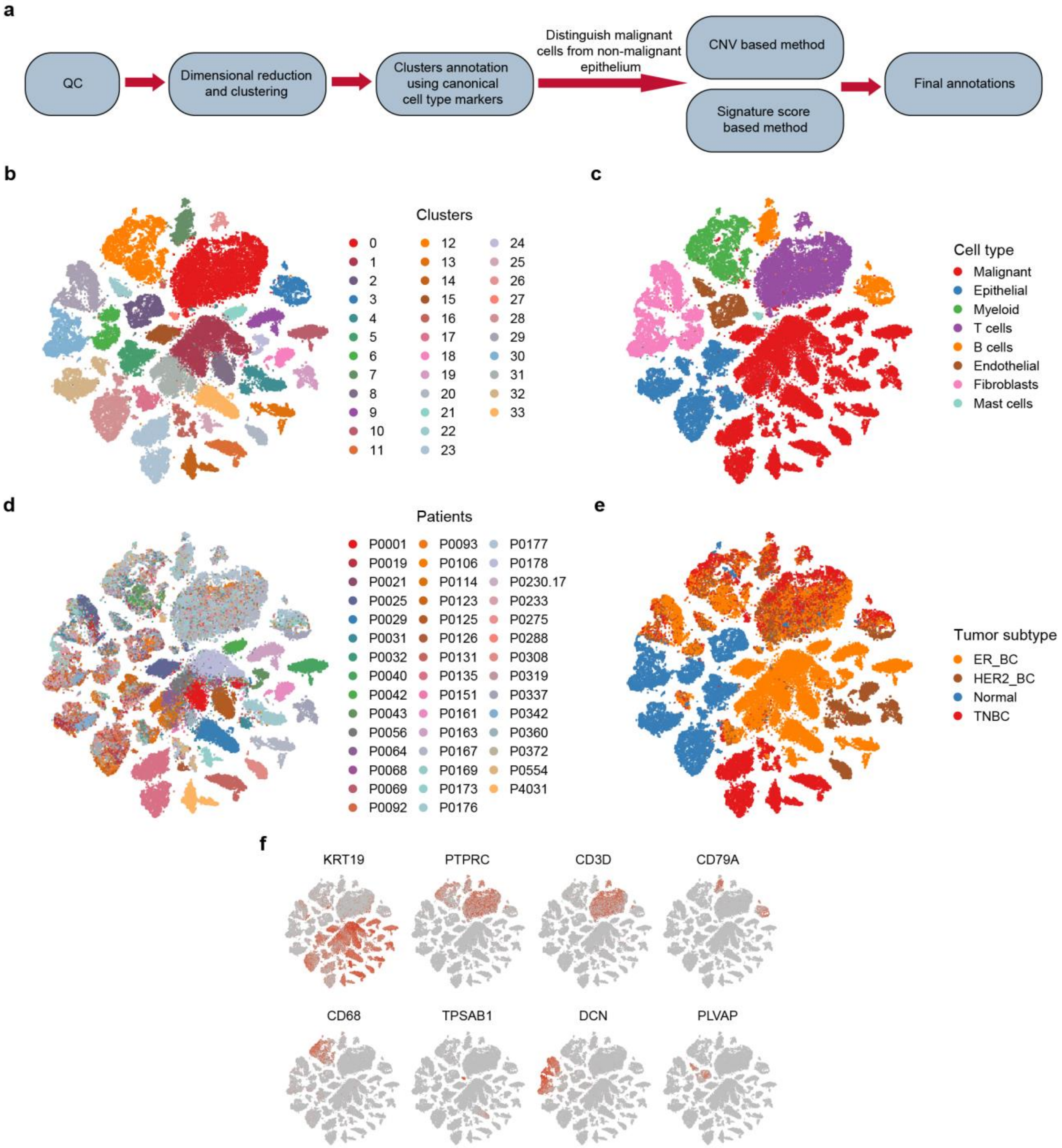

Figure S1. Identification of main cell lineages. **(a)** Schematics of uniform pipelines for cell lineage identification. **(b-e)** t-SNE visualization of cell clusters (**b**), cell types (**c**), patients (**d**) and tumour subtypes (**e**) for the BRCA dataset. **(f)** t-SNE plots as in (**b**) coloured by expression levels of representative markers of main cell lineages. The color key from grey to red indicates relative expression levels from low to high.

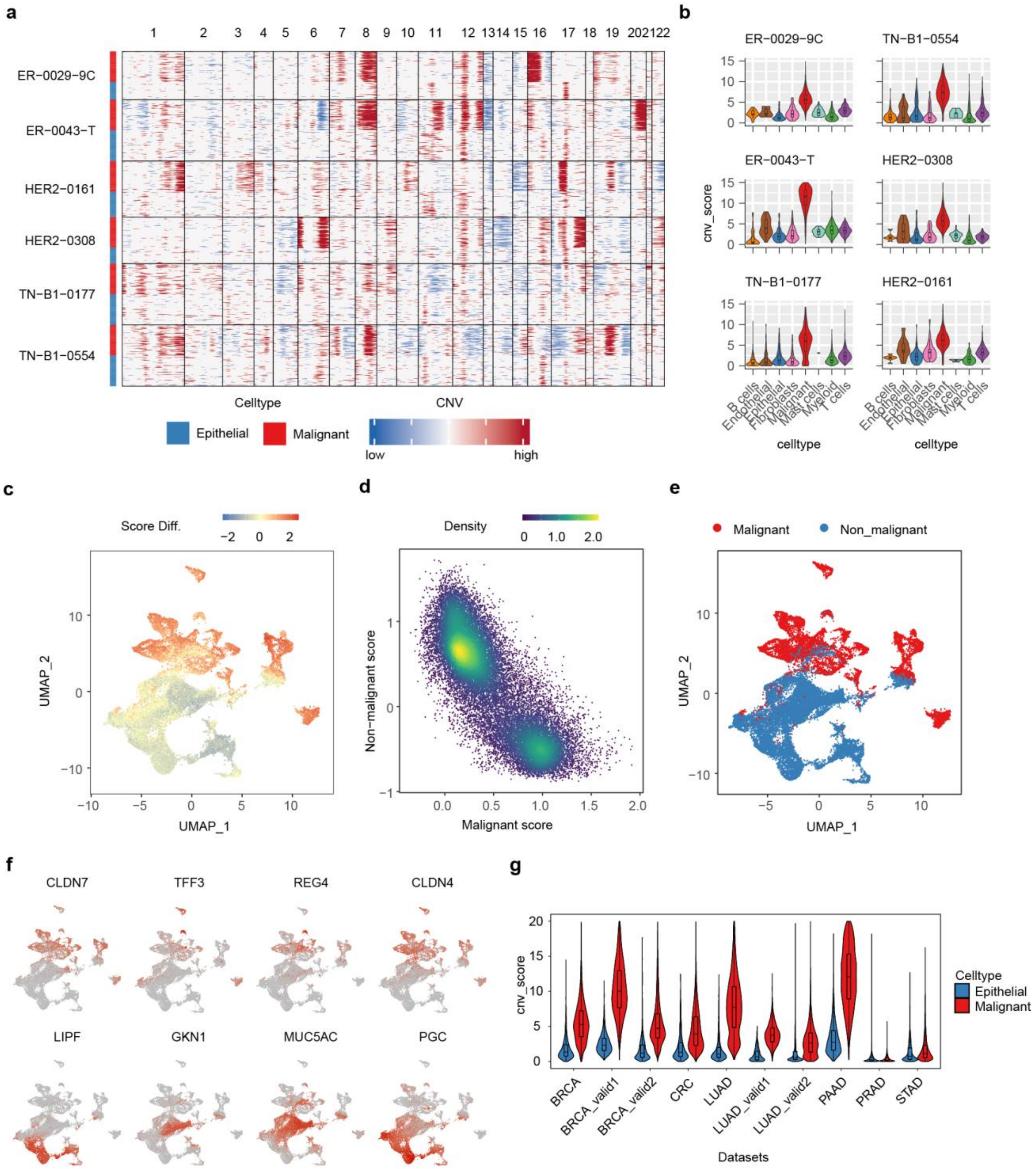

Figure S2. Distinguish malignant cells from non-malignant epithelium. **(a)** and **(b)** Using estimated CNVs to identify malignant cells in BRCA. **(a)** Heatmap showing large-scale CNVs of epithelial compartment (malignant and normal epithelial cells) from 6 representative BRCA samples, covering estrogen receptor (ER)$^{+}$, HER2^{+} and triple-negative (TN) breast cancers. The normalized CNV levels were shown, the red color represents high CNV level and blue represents low CNV level. **(b)** Violin and box plots showing distributions of CNV scores among different cell types from the same samples as in (**a**). **(c-f)** Using score-based method to identify malignant cells in STAD. **(c)** UMAP plot of the epithelial compartment in STAD, color-coded according to malignant score minus non-malignant score. **(d)** Scatter plot displaying the distribution of malignant scores (x-axis) and non-malignant scores (y-axis).

Each point corresponds to a cell and is color-coded to reflect density. **(e)** UMAP plot of the classification of malignant and non-malignant cells. **(f)** UMAP plot showing expression of eight representative genes with differential expression. **(g)** CNV signals of finally identified malignant cells compared with normal epithelial cells.

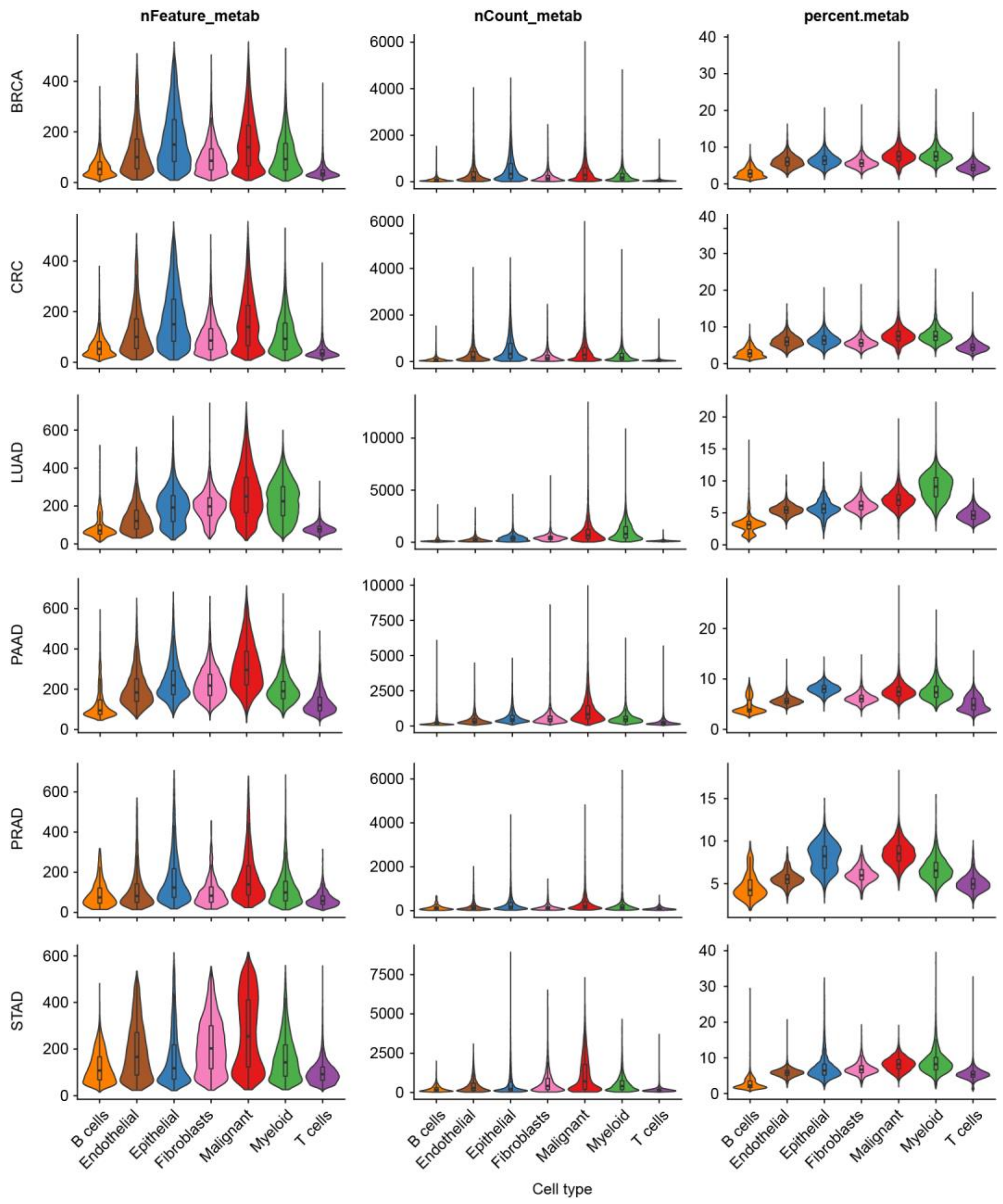

Figure S3. Overall characteristics of metabolic gene expression. Violin and box plots showing distributions of "nFeature_metab", "nCount_metab" and "percent.metab" among different cell types in main datasets. nFeature_metab, number of expressed metabolic genes; nCount_metab, number of UMIs; percent.metab, percent of metabolic reads.

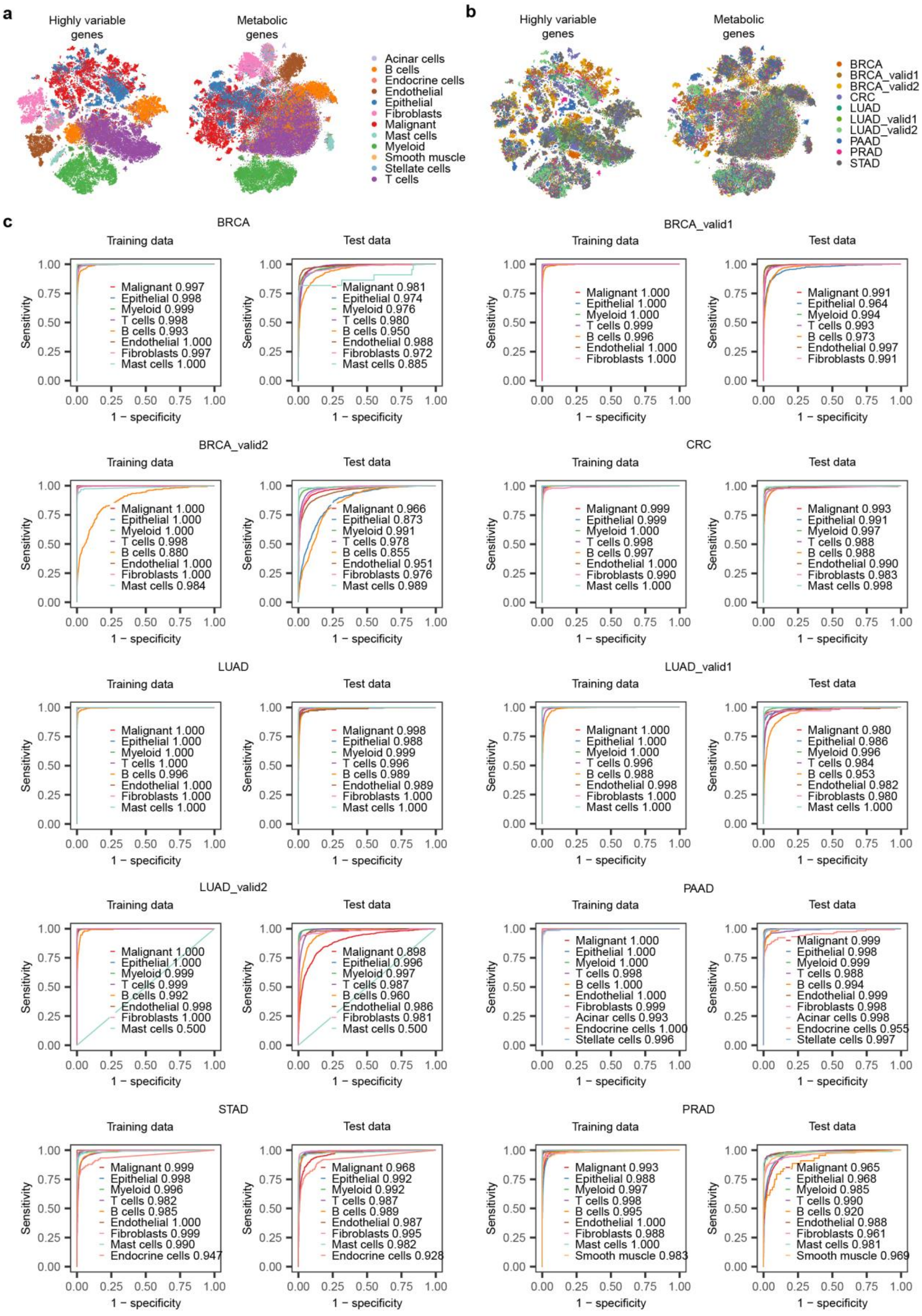
a
Highly variable genes
Metabolic genes
Acinar cells
B cells
Endocrine cells
Endothelial
Epithelial
Fibroblasts
Malignant
Mast cells
Myeloid
Smooth muscle
Stellate cells
T cells
b
Highly variable genes
Metabolic genes
BRCA
BRCA_valid1
BRCA_valid2
CRC
LUAD
LUAD_valid1
LUAD_valid2
PAAD
PRAD
STAD
c
BRCA
Training data
Malignant 0.997
Epithelial 0.998
Myeloid 0.999
T cells 0.998
B cells 0.993
Endothelial 1.000
Fibroblasts 0.997
Mast cells 1.000
Test data
Malignant 0.981
Epithelial 0.974
Myeloid 0.976
T cells 0.980
B cells 0.950
Endothelial 0.988
Fibroblasts 0.972
Mast cells 0.885
BRCA_valid1
Training data
Malignant 1.000
Epithelial 1.000
Myeloid 1.000
T cells 0.999
B cells 0.996
Endothelial 1.000
Fibroblasts 1.000
Test data
Malignant 0.991
Epithelial 0.964
Myeloid 0.994
T cells 0.993
B cells 0.973
Endothelial 0.997
Fibroblasts 0.991
BRCA_valid2
Training data
Malignant 1.000
Epithelial 1.000
Myeloid 1.000
T cells 0.998
B cells 0.880
Endothelial 1.000
Fibroblasts 1.000
Mast cells 0.984
Test data
Malignant 0.966
Epithelial 0.873
Myeloid 0.991
T cells 0.978
B cells 0.855
Endothelial 0.951
Fibroblasts 0.976
Mast cells 0.989
CRC
Training data
Malignant 0.999
Epithelial 0.999
Myeloid 1.000
T cells 0.998
B cells 0.997
Endothelial 1.000
Fibroblasts 0.990
Mast cells 1.000
Test data
Malignant 0.993
Epithelial 0.991
Myeloid 0.997
T cells 0.988
B cells 0.988
Endothelial 0.990
Fibroblasts 0.983
Mast cells 0.998
LUAD
Training data
Malignant 1.000
Epithelial 1.000
Myeloid 1.000
T cells 1.000
B cells 0.996
Endothelial 1.000
Fibroblasts 1.000
Mast cells 1.000
Test data
Malignant 0.998
Epithelial 0.988
Myeloid 0.999
T cells 0.996
B cells 0.989
Endothelial 0.989
Fibroblasts 1.000
Mast cells 1.000
LUAD_valid1
Training data
Malignant 1.000
Epithelial 1.000
Myeloid 1.000
T cells 0.998
B cells 0.988
Endothelial 0.998
Fibroblasts 1.000
Mast cells 1.000
Test data
Malignant 0.980
Epithelial 0.986
Myeloid 0.996
T cells 0.984
B cells 0.953
Endothelial 0.982
Fibroblasts 0.980
Mast cells 1.000
LUAD_valid2
Training data
Malignant 1.000
Epithelial 1.000
Myeloid 0.999
T cells 0.999
B cells 0.992
Endothelial 0.998
Fibroblasts 1.000
Mast cells 0.500
Test data
Malignant 0.898
Epithelial 0.996
Myeloid 0.997
T cells 0.987
B cells 0.960
Endothelial 0.986
Fibroblasts 0.981
Mast cells 0.500
PAAD
Training data
Malignant 1.000
Epithelial 1.000
Myeloid 1.000
T cells 0.998
B cells 1.000
Endothelial 1.000
Fibroblasts 0.999
Acinar cells 0.993
Endocrine cells 1.000
Stellate cells 0.996
Test data
Malignant 0.999
Epithelial 0.998
Myeloid 0.999
T cells 0.988
B cells 0.994
Endothelial 0.999
Fibroblasts 0.998
Acinar cells 0.998
Endocrine cells 0.955
Stellate cells 0.997
STAD
Training data
Malignant 0.999
Epithelial 0.998
Myeloid 0.996
T cells 0.982
B cells 0.985
Endothelial 1.000
Fibroblasts 0.999
Mast cells 0.990
Endocrine cells 0.947
Test data
Malignant 0.968
Epithelial 0.992
Myeloid 0.992
T cells 0.987
B cells 0.989
Endothelial 0.987
Fibroblasts 0.995
Mast cells 0.982
Endocrine cells 0.928
PRAD
Training data
Malignant 0.993
Epithelial 0.988
Myeloid 0.997
T cells 0.998
B cells 0.995
Endothelial 1.000
Fibroblasts 0.988
Mast cells 1.000
Smooth muscle 0.983
Test data
Malignant 0.965
Epithelial 0.968
Myeloid 0.985
T cells 0.990
B cells 0.920
Endothelial 0.988
Fibroblasts 0.961
Mast cells 0.981
Smooth muscle 0.969
Sensitivity
1 − specificity
0.00
0.25
0.50
0.75
1.00

Figure S4. Robust metabolic differences across major cell lineages. (a) t-SNE plots showing major cell lineages for combined datasets based on highly variable genes and metabolic genes, respectively. **(b)** Similar as in (**a**), color-coded according to the datasets to which they belong. **(c)** L1-regularized linear regression (using only metabolic genes) was trained on a subset of donors and tested on a separate set of donors for each dataset, respectively. Stated numbers report the AUC for the indicated population.

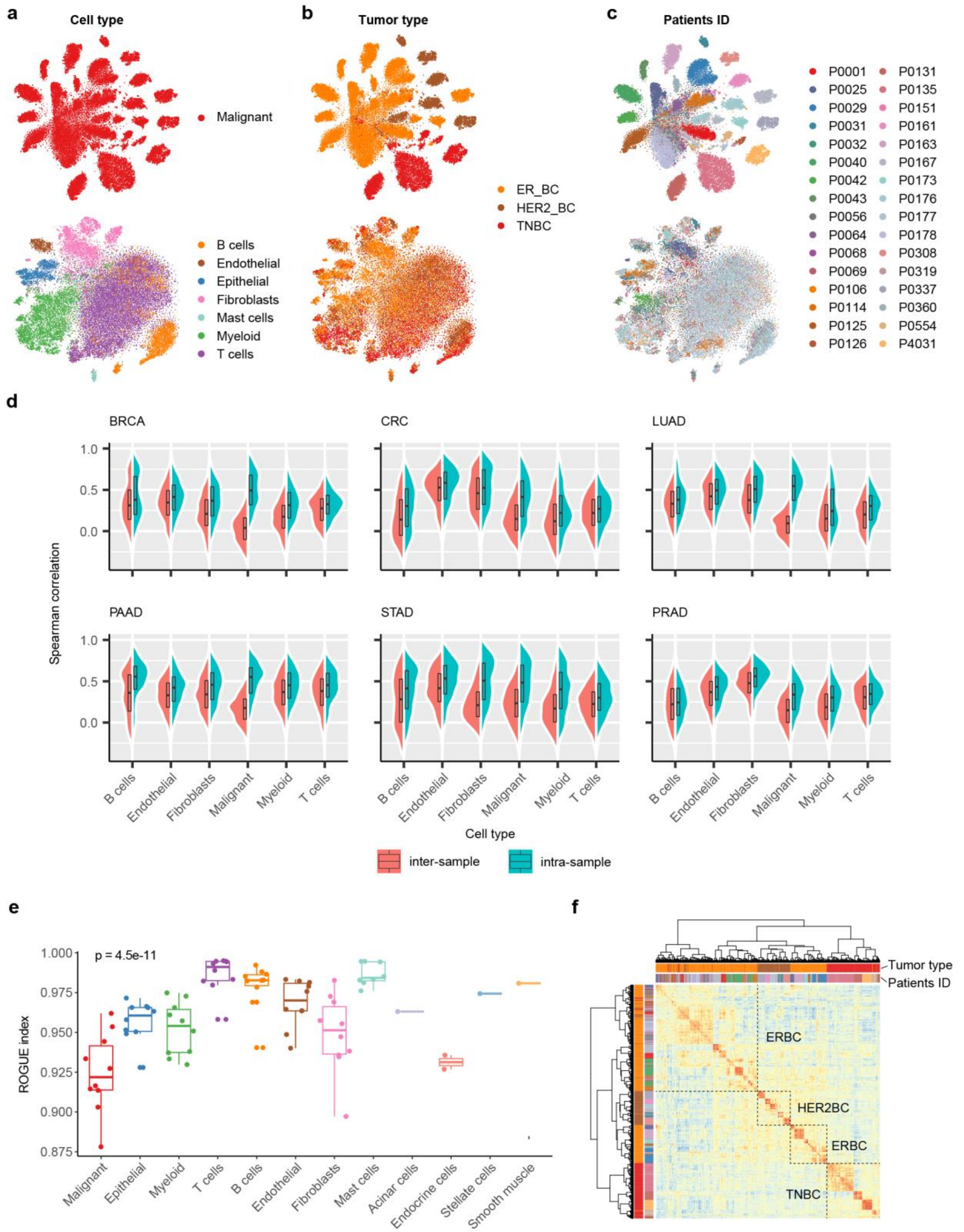

Figure S5. Malignant cells show higher metabolic heterogeneity than non-malignant cells. (a) t-SNE plots showing major cell lineages for malignant cells (top) and non-malignant cells (bottom) in BRCA, respectively. **(b)** Similar as in (**a**), color-coded according to the tumour subtype. **(c)** Similar as in (**a**), color-coded according to individual patients. **(d)** Distributions of cell-to-cell metabolic similarity of 500 randomly selected pairs of cells from the same sample (intra-sample) or from different samples (inter-sample). **(e)** Box plot showing the cell purity of

metabolic gene profiles for each major cell lineage by ROGUE. *P* value were calculated using one-way ANOVA test. **(f)** Clustered heatmap showing cell-to-cell metabolic similarity between malignant cells in the BRCA dataset.

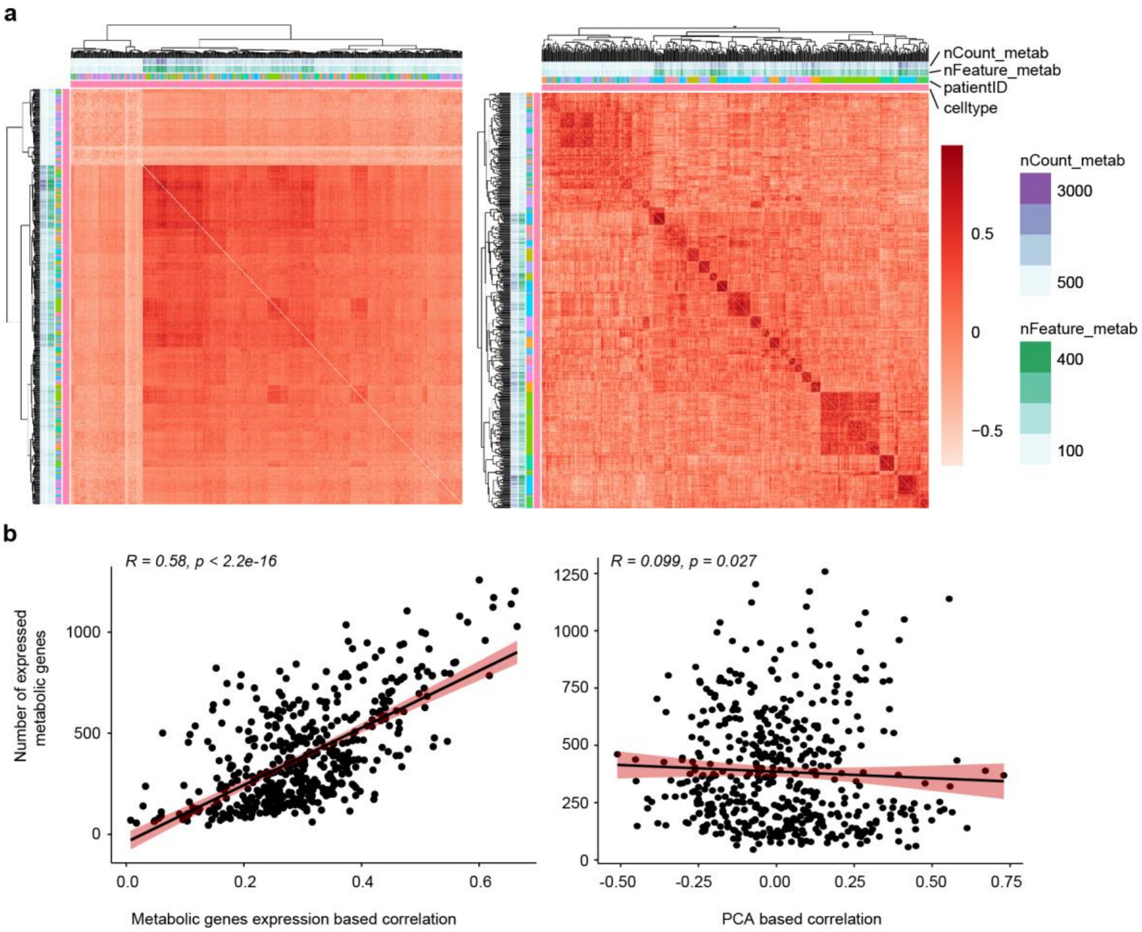

Figure S6. Quantification of cell-to-cell metabolic similarity in CRC dataset based on different methods. **(a)** Heatmap showing the cell-to-cell metabolic similarity of malignant cells based on correlation of metabolic gene expressions (left) and principal components (right). **(b)** Scatter plots of the number of expressed metabolic genes (y-axis) and cell-to-cell metabolic similarity (x-axis) based on different methods, 500 pairs of malignant cells are shown. Spearman correlation coefficients were reported.

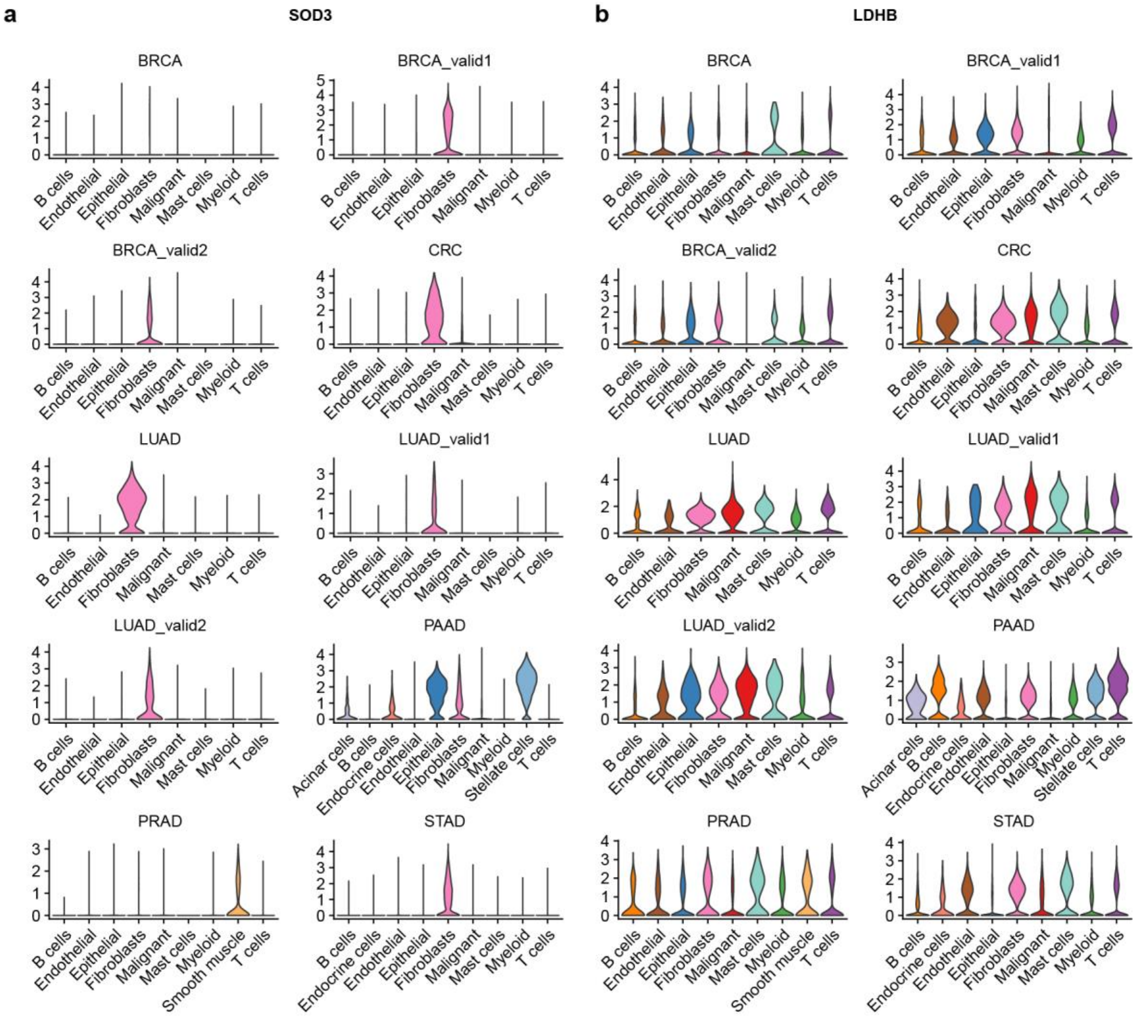

Figure S7. Representative genes showing prominent heterogeneity across cancer types. (a) and **(b)** Violin plots showing the expression of *SOD3* **(a)** and *LDHB* **(b)** across cell types in each dataset.

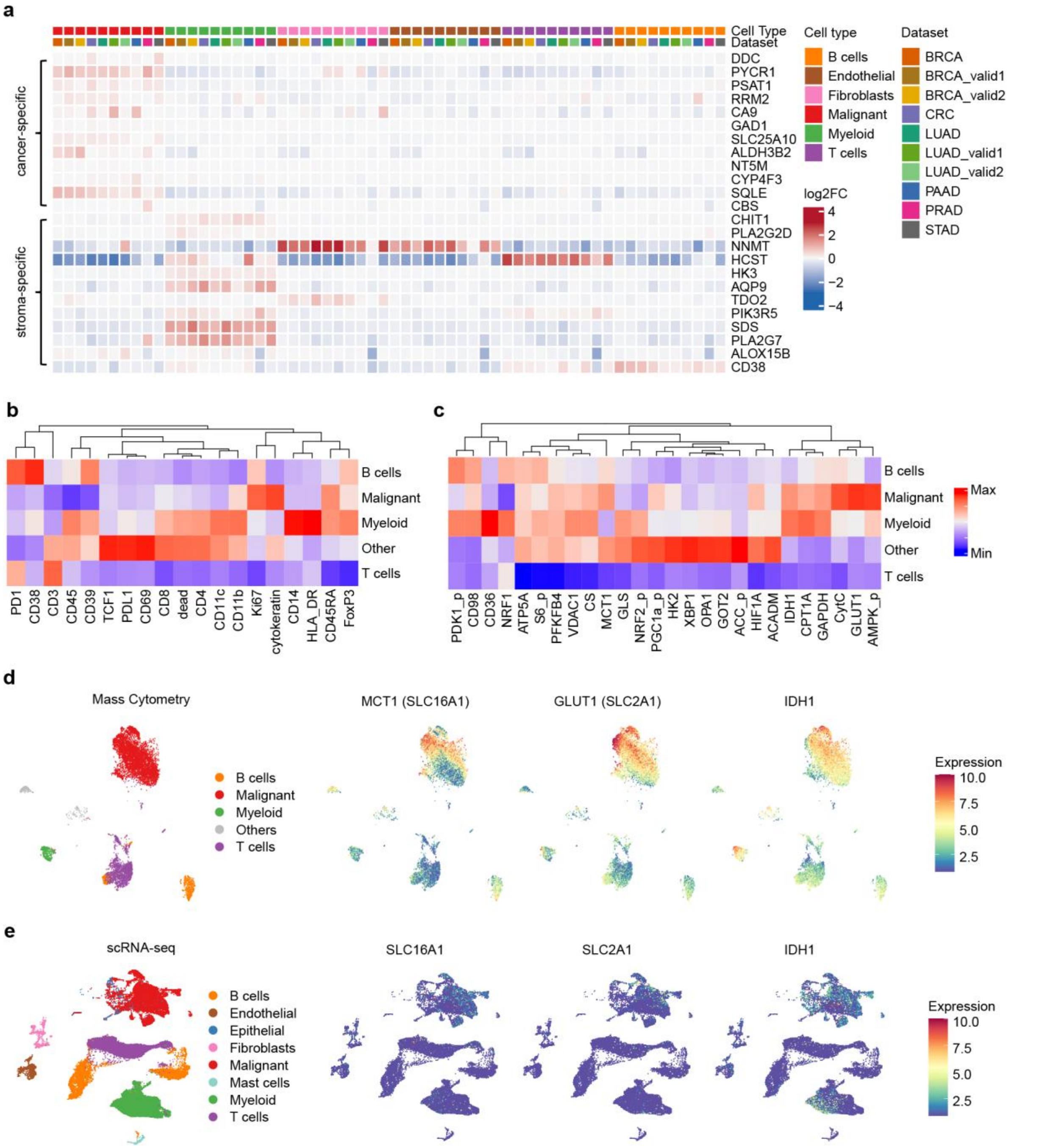

Figure S8. Validation of metabolic signature genes of malignant cells using mass cytometry data of CRC. (a) Validation of cell type-specificity of metabolic genes identified by Rohatgi et al. **(b)** and **(c)** Heatmap showing the expression of major lineage markers **(b)** and metabolic features **(c)** across cell populations in mass cytometry data of CRC. **(d)** UMAP plots showing the major cell lineages and representative metabolic genes of malignant cells in mass cytometry data of CRC. **(e)** Similar plot as in **(d)** for scRNA-seq data of CRC.

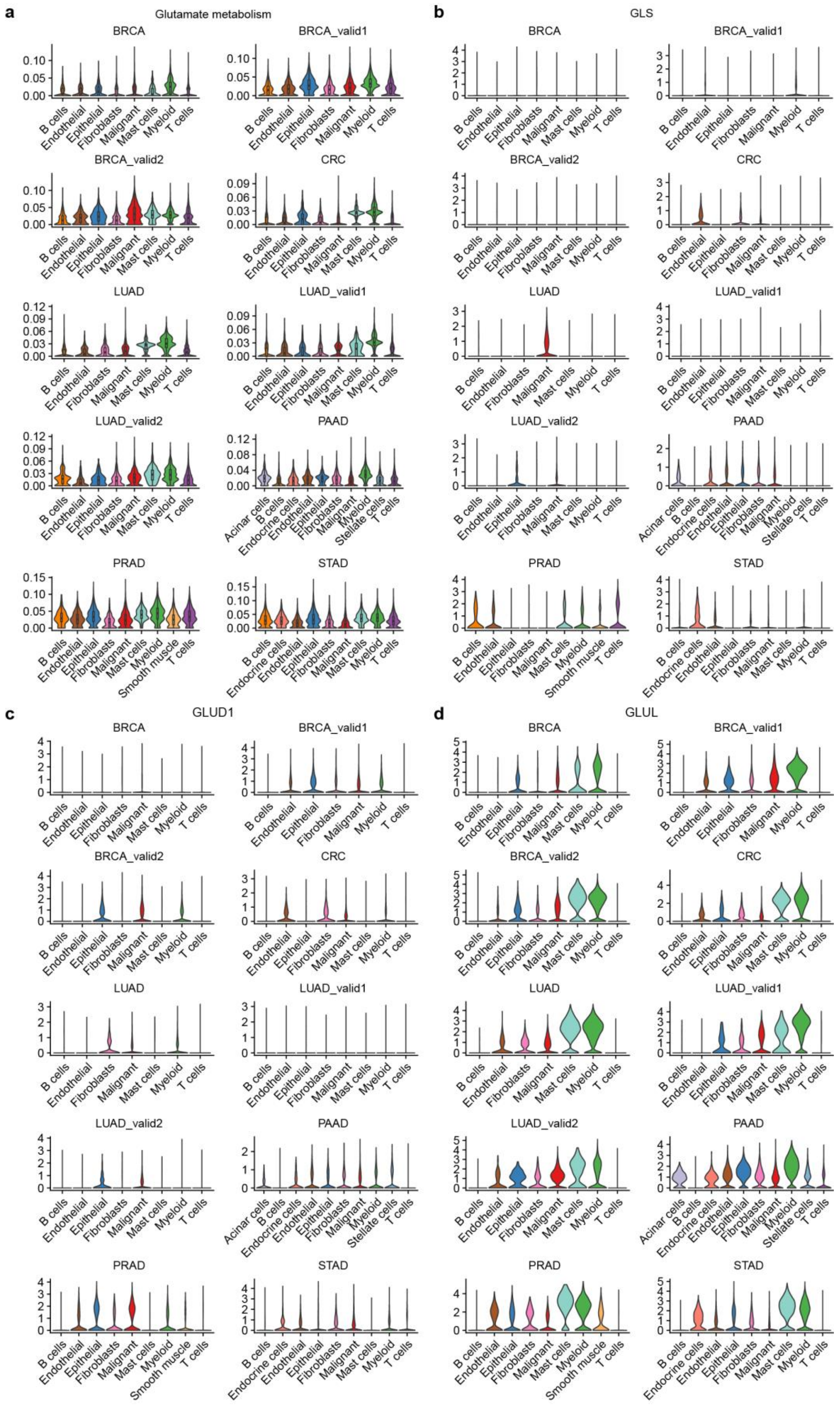
a
Glutamate metabolism
BRCA
BRCA_valid1
BRCA_valid2
CRC
LUAD
LUAD_valid1
LUAD_valid2
PAAD
PRAD
STAD
b
GLS
c
GLUD1
d
GLUL
B cells
Endothelial
Epithelial
Fibroblasts
Malignant
Mast cells
Myeloid
T cells
Acinar cells
Endocrine cells
Stellate cells
Smooth muscle

Figure S9. High level of glutamate metabolism in myeloid cells. **(a-d)** Violin and box plots showing the level of glutamate metabolism **(a)** and expression of core enzyme genes **(b-d)** across cell types in each dataset.

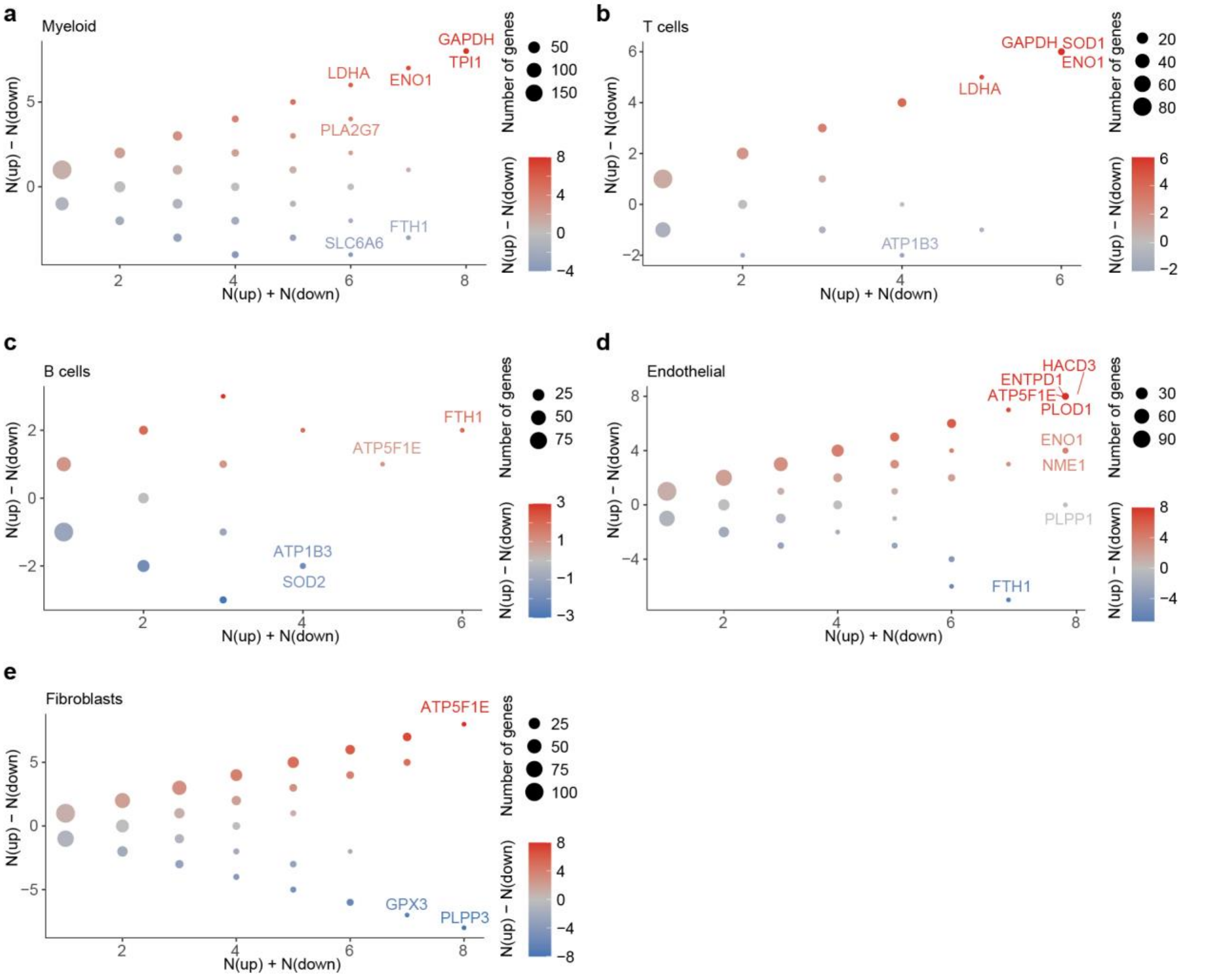

Figure S10. Gene signatures of metabolic reprogramming of non-malignant cells. **(a-e)** Similar plots as in Fig. 3a for non-malignant cell types.

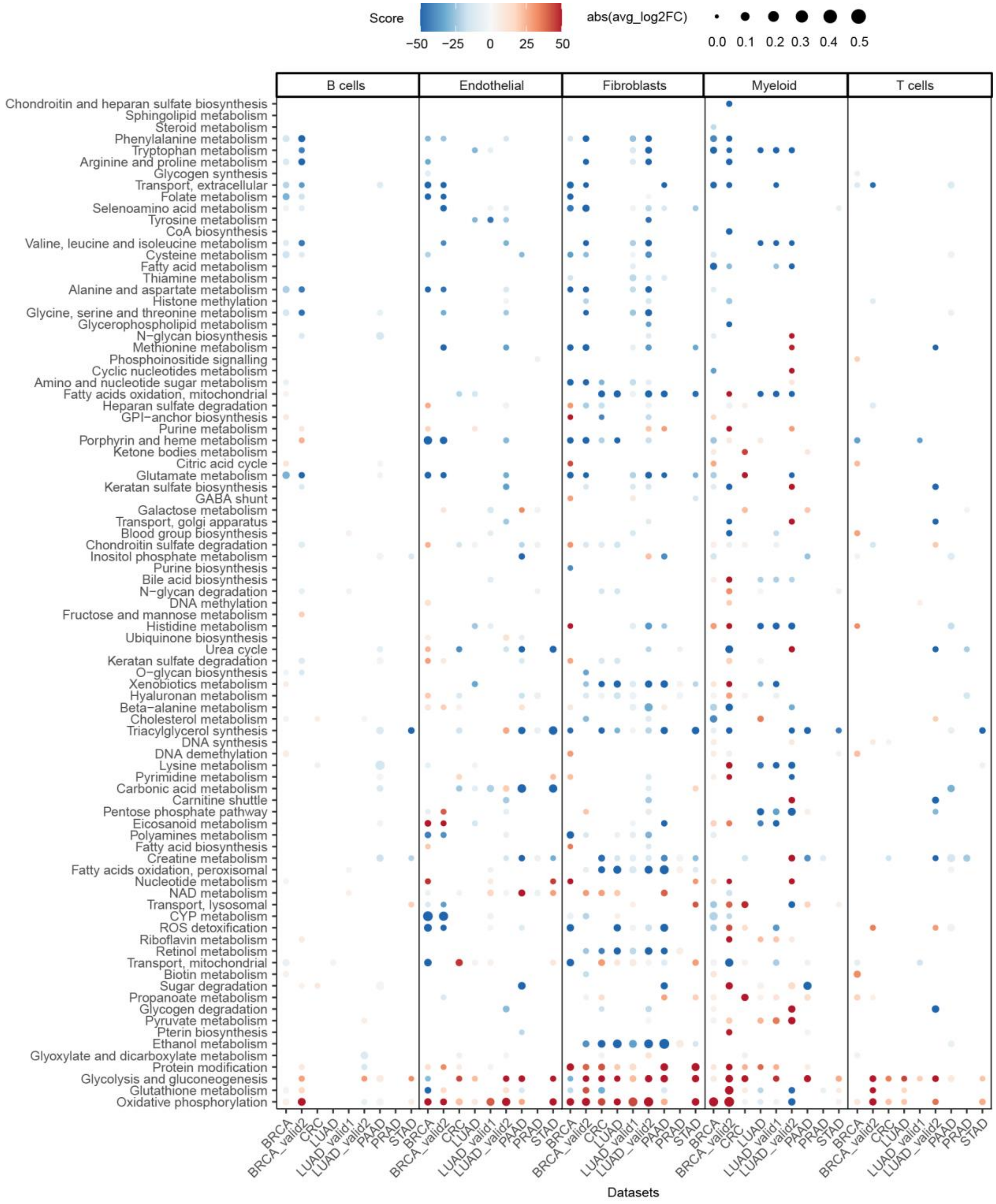

Figure S11. Pathway signatures of metabolic reprogramming of non-malignant cells. Similar plot as in Fig. 3c for non-malignant cell types.

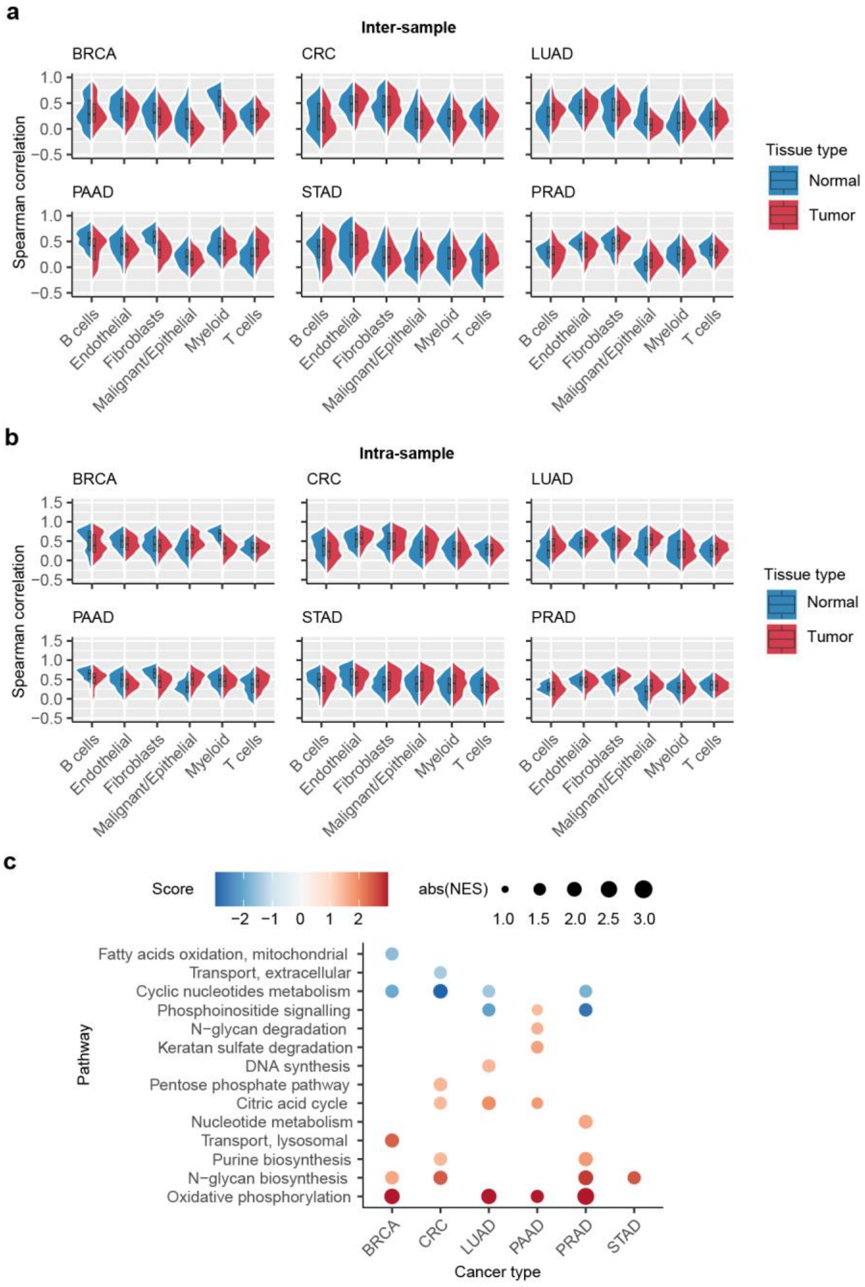

Figure S12. Alteration of metabolic similarity between tumours and normal tissues. (a) Comparison of inter-sample cell-to-cell metabolic similarity of 500 randomly selected cell pairs in tumours and normal tissues. **(b)** Comparison of intra-sample cell-to-cell metabolic similarity of 500 randomly selected cell pairs in tumours and normal tissues. **(c)** Dot plot showing the pathway signatures of individual-level metabolic reprogramming. Dot is coloured according to differential score, and dot size indicates absolute value of normalized enrichment score of GSEA.

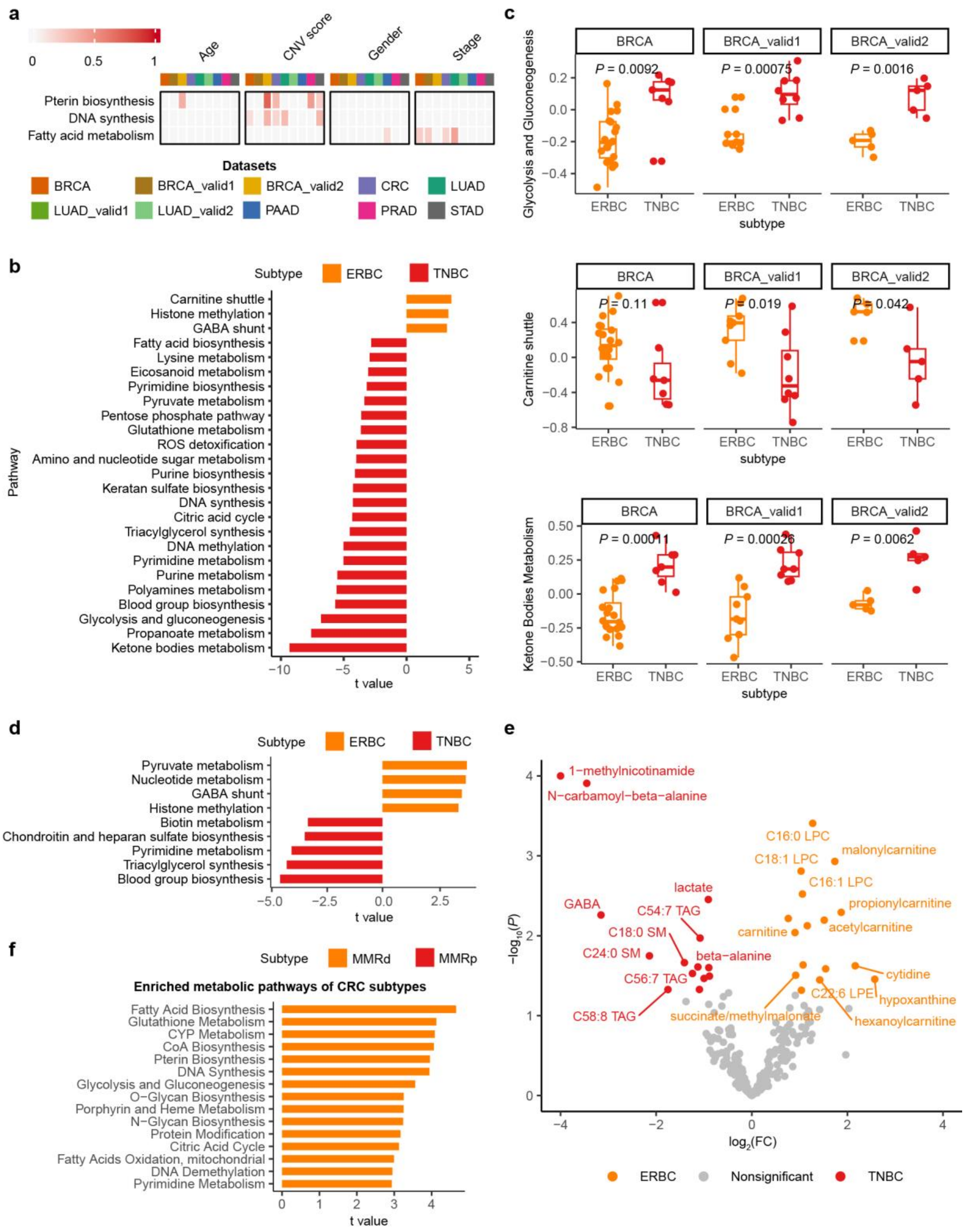

Figure S13. Intertumour metabolic heterogeneity of malignant cells. **(a)** Heatmap showing the proportion of variance explained (PVE) of metabolic pathways by individual clinical metrics. Color represents the PVE, which is measured using the adjusted R^2 from a linear model. *P* value was calculated by unpaired two-tailed Student's t test in the linear model. Only significant associations with PVE > 0.1 and *P* value < 0.05 are coloured, and insignificant associations are left in blank. Only pathways with significant association in no less than 4 datasets were shown. **(b)** Bar plot showing the enriched pathways of BRCA subtypes. Pathway scores were calculated using GSVA and

significantly enriched pathways with FDR < 0.05 (unpaired two-tailed Student's t test, BH correction) are highlighted in orange (higher in ERBC) and red (higher in TNBC). **(c)** Box plots showing the levels of representative pathways between ERBC and TNBC in different datasets of BRCA. *P* value was calculated by unpaired two-tailed Student's t test. **(d)** Bar plot showing the enriched pathways (Student's t test, FDR < 0.05) of cell lines derived from different BRCA subtypes. **(e)** Volcano plot of metabolites showing significant changes ($P <$ 0.05, Student's t test) between cell lines derived from different BRCA subtypes. The x-axis shows $\log_2$ fold change ($\log_2$(FC)), and the y-axis shows $-\log_{10}$(P value). **(f)** Bar plot showing the enriched pathways of CRC subtypes.

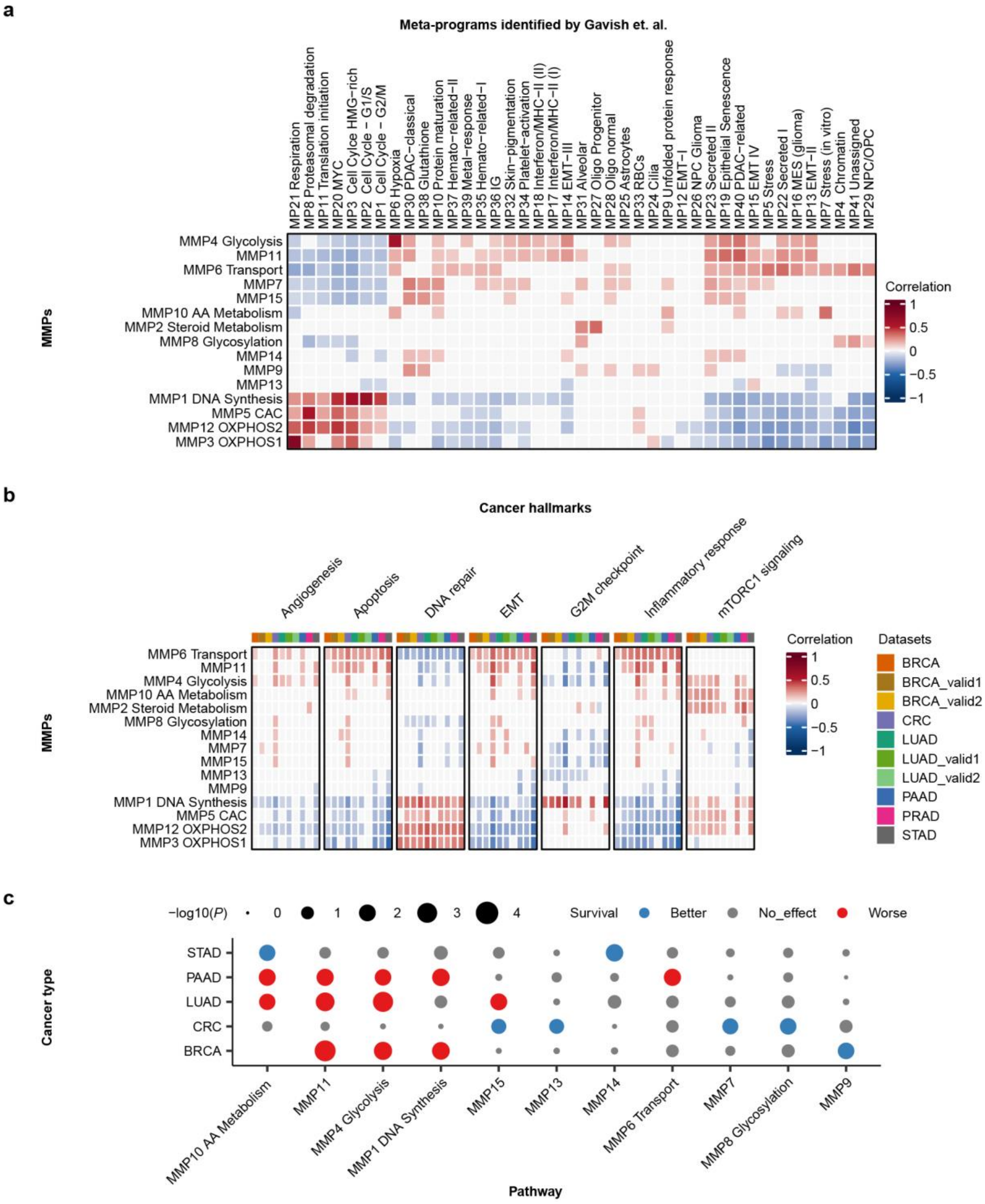

Figure S14. Associations between malignant MMPs and other factors. **(a)** Heatmap showing the correlations between MMPs and meta-programs identified by Gavish et al. Pearson correlation coefficients were calculated within each tumour and then averaged across all tumours. Significant correlations (Mean Pearson correlation coefficient > 0.1 or < -0.1, and FDR < 0.05) are coloured according to the correlation coefficients. **(b)** Heatmap showing the correlations between MMPs and cancer hallmarks. Pearson correlation coefficients were calculated within each tumour and then averaged across tumours within each dataset. Significant correlations (Mean Pearson correlation coefficient > 0.1 or < -0.1, and FDR < 0.05) are coloured according to the correlation coefficients. **(c)** Clinical associations of MMPs with patient overall survival in TCGA. Significant associations (Cox regression, *P*

value < 0.05) are coloured with red or blue. Red indicates that higher expression of a MMP is associated with worse prognosis and blue indicates the opposite. Dot size indicates -log10(*P* value).

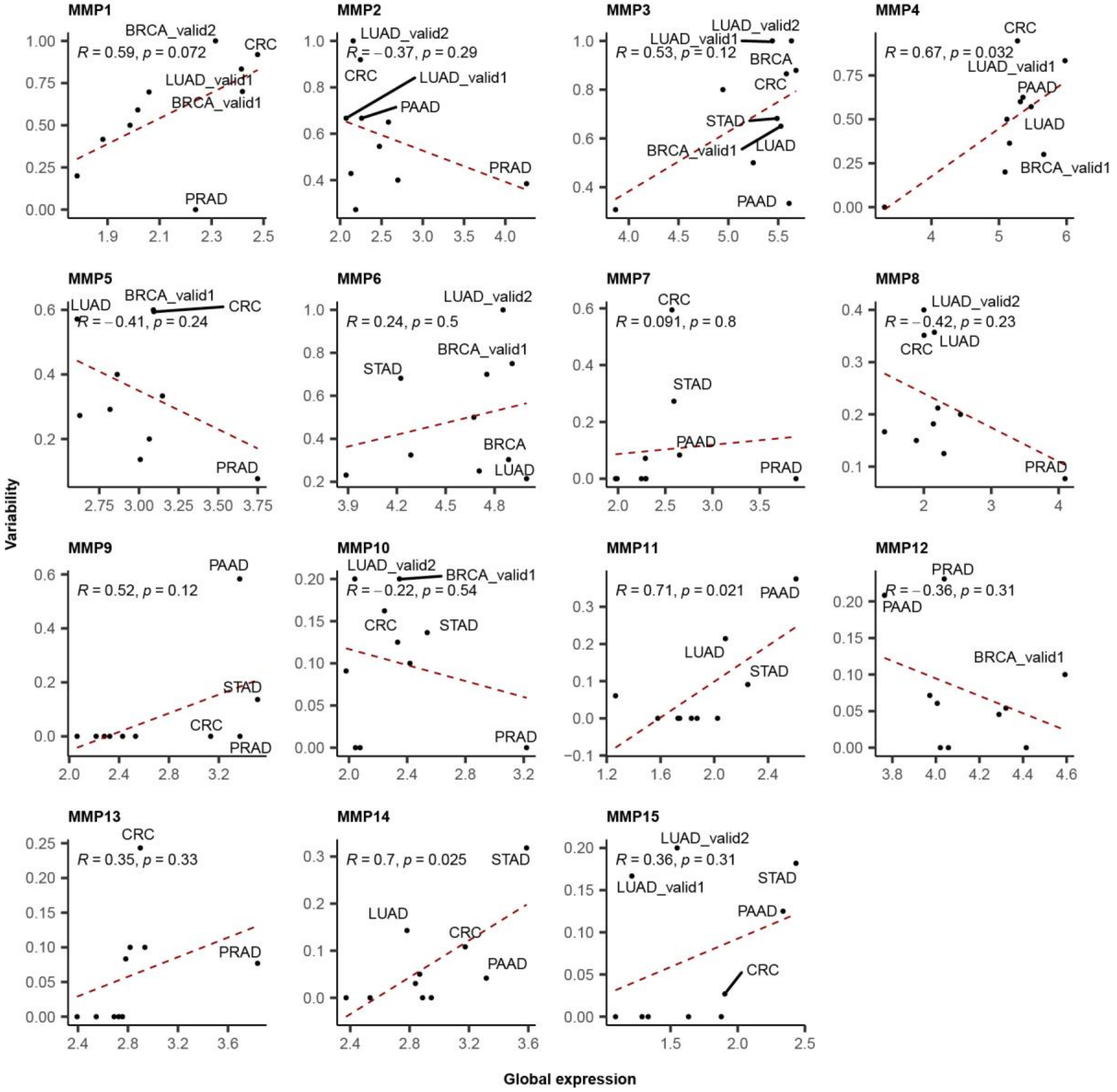

Figure S15. Global (mean) expression vs. variability of MMP expression across different datasets. Each panel shows global expression (x-axis) and variability of expression (y-axis) of a specific MMP across all datasets (dots), along with a Pearson correlation, *P* value and labels for selected cancer types. Global expression is defined as the mean expression of the MMP genes across malignant cells from all samples of a specific dataset. Variability of the MMP in a given dataset is defined as the fraction of tumours of that dataset in which at least one of the robust NMF programs was assigned to the MMP.

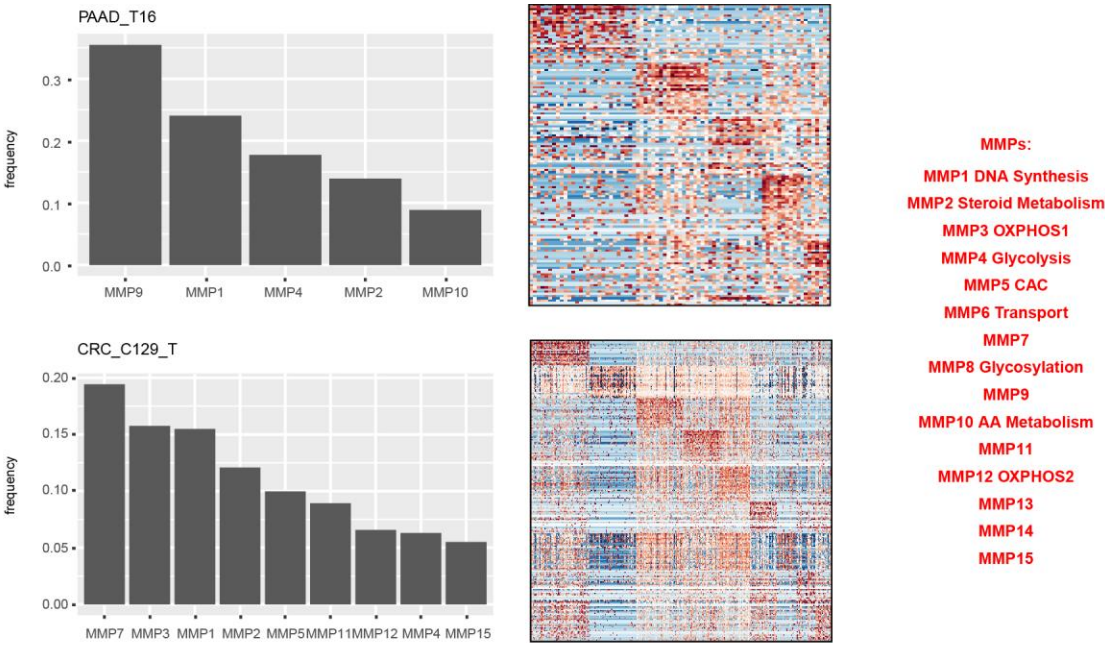

Figure S16. MMP distribution in representative samples of PAAD and CRC. Bar plots show the frequency of cells assigned to specific MMP. Heatmaps show the expression of MMP genes (rows) across malignant cells (columns) in the sample.

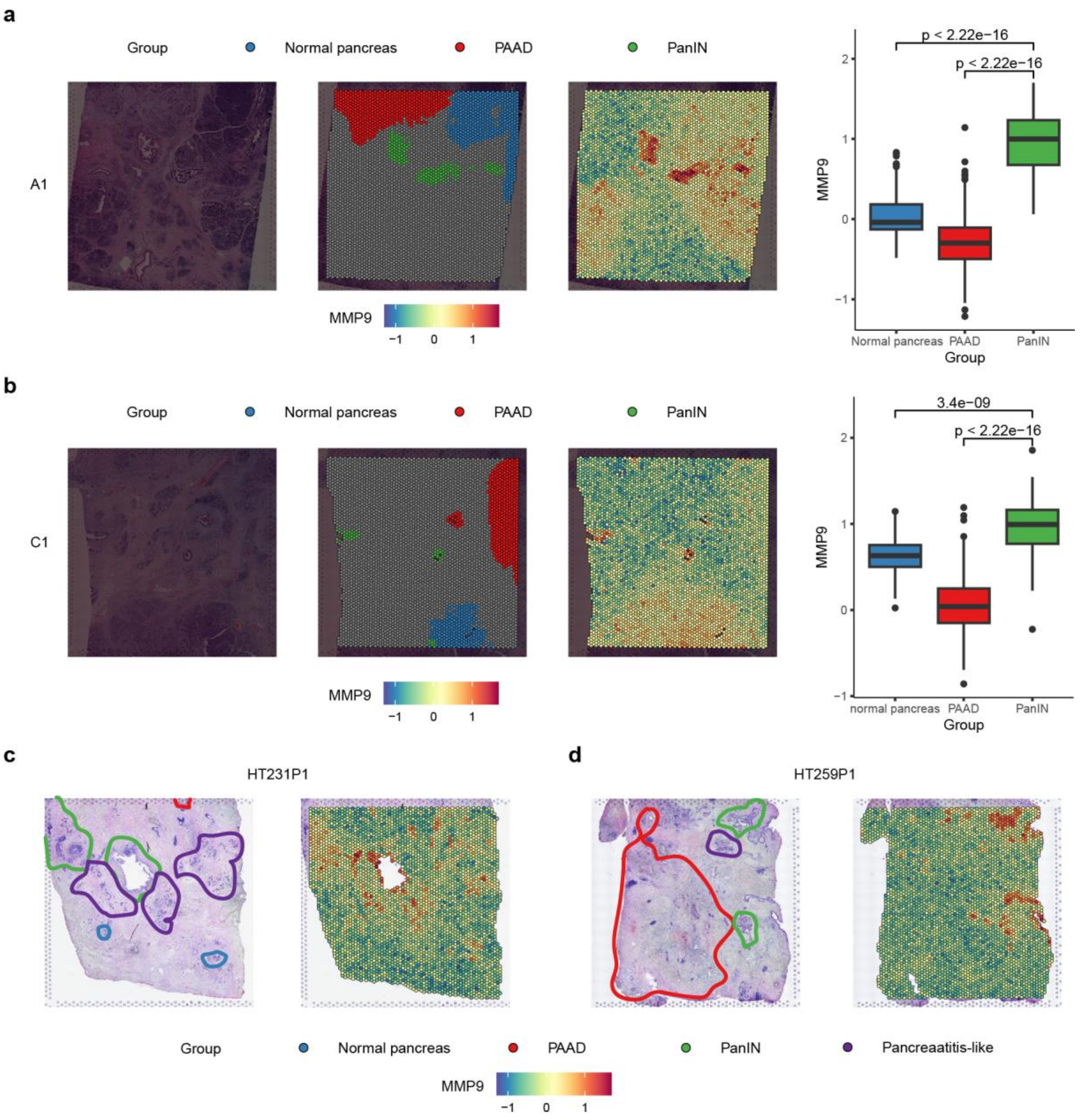

Figure S17. MMP9 expression in ST data of PAAD. (a) and (b) ST data of two samples from our lab (unpublished). Left, Visium images show the haematoxylin and eosin (H&E) staining, spots annotations and MMP9 scores, respectively. Right, box plot shows the distribution of MMP9 scores in normal pancreas, PAAD and PanIN spots. Two-sided Wilcoxon rank sum test *P* values were shown. **(c) and (d)** ST data of two samples from a previous study. Left, as spots annotations could not be obtained, regions were labelled on Visium image according to the figures from the original study. Right, Visium image shows MMP9 scores.

a

b

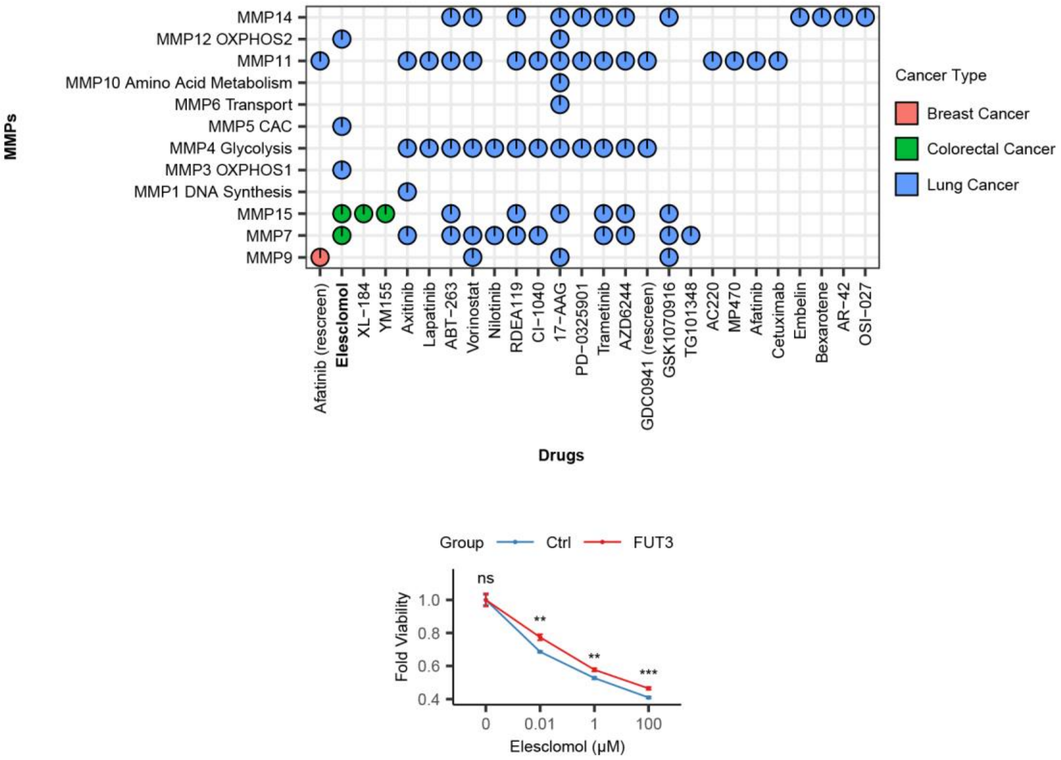

Figure S18. MMPs and drug sensitivity. (a) Scatter pie plot shows all significant correlations between MMPs and drug sensitivity for cancer types investigated in this study. **(b)** The effect of *FUT3* overexpression on drug response to elesclomol at 24 hours in HCT116 cells. Data are represented as mean ± SEM. Compared to negative control, ns means not significant; $^{**}P < 0.01$; $^{***}P < 0.001$.

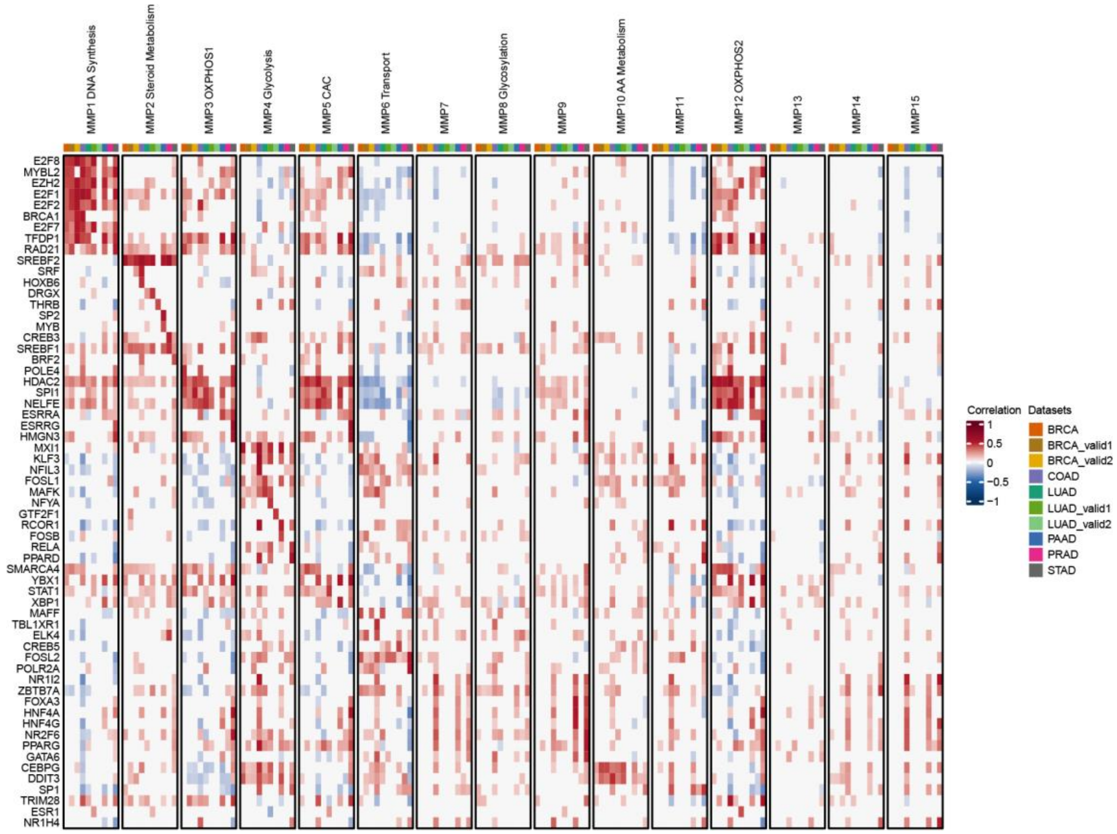

Figure S19. MMP regulators for malignant cells. Heatmap showing the correlations between regulon scores and MMP scores. Pearson correlation coefficients were calculated within each tumour and then averaged across tumours within each dataset. Regulons with adjusted *P* value (both Pearson and Spearman) < 0.05 and mean Pearson correlation coefficient > 0.4 or < -0.4 were kept. If more than three regulons exist, then only top three were retained. Finally, regulons for all combinations of datasets and MMPs were merged and plotted.

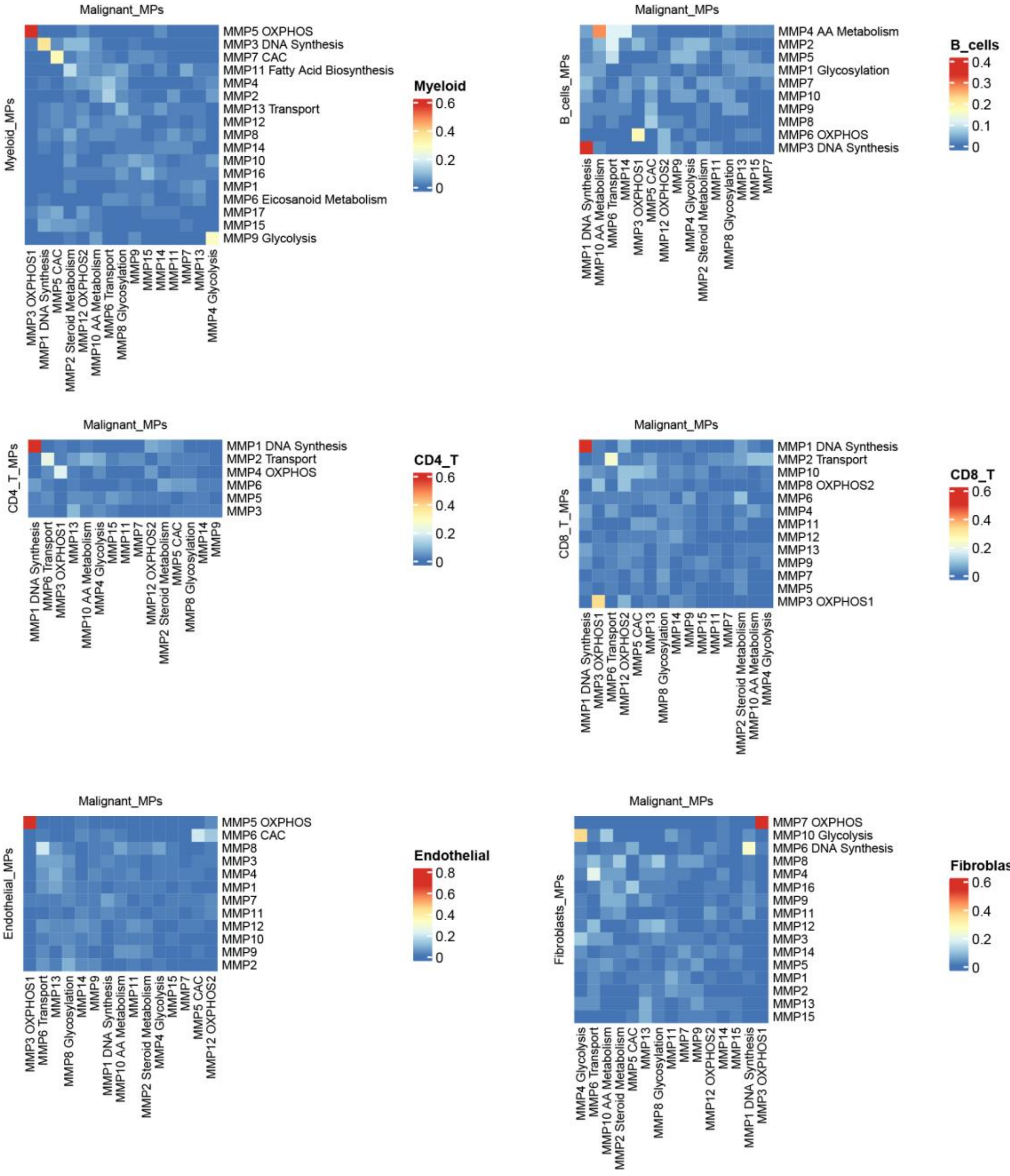

Figure S20. Similarity between malignant MMPs and non-malignant MMPs. Heatmap shows Jaccard similarity between MMPs of malignant cells (x-axis) and non-malignant cell types (y-axis).

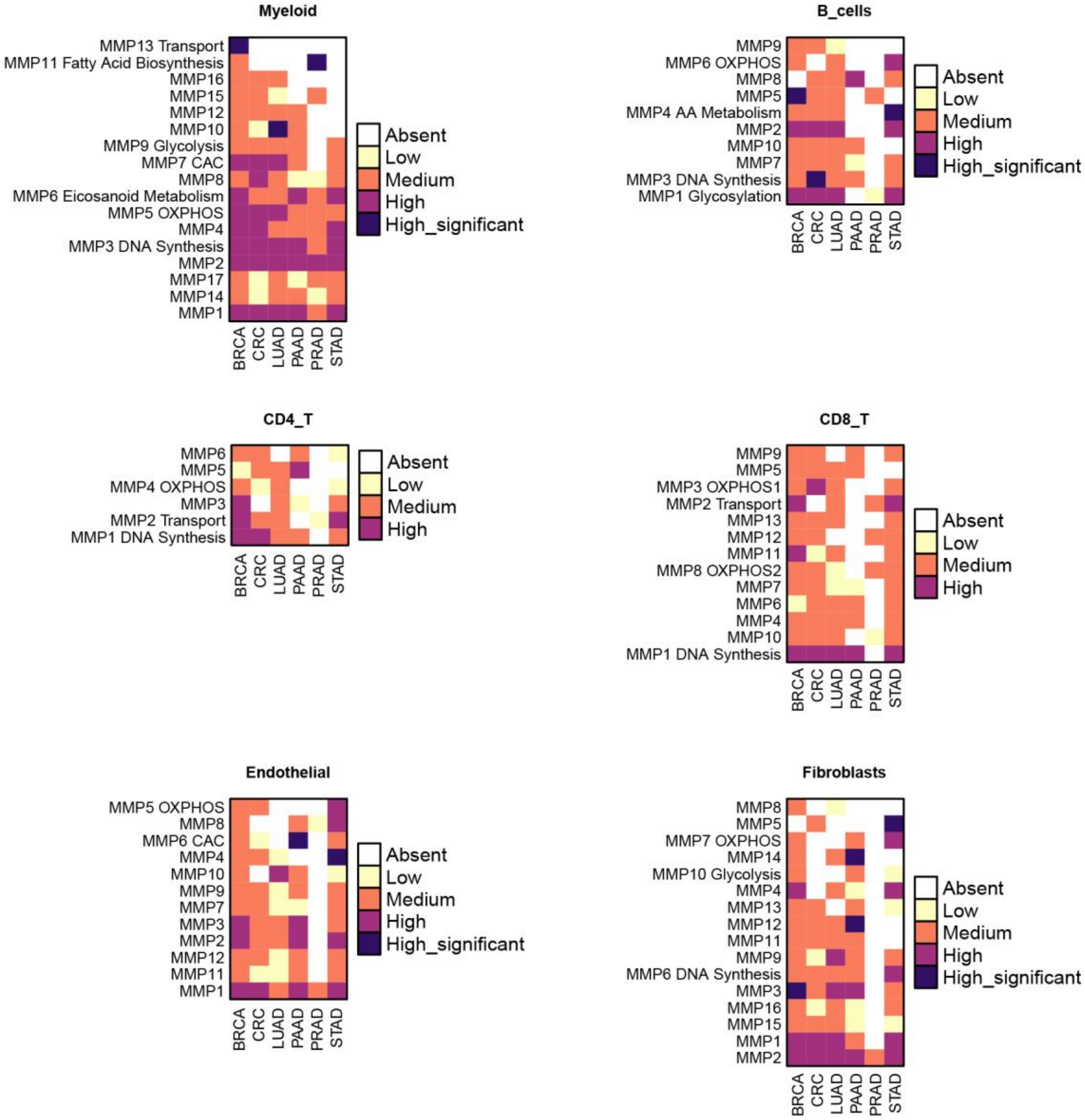

Figure S21. Abundance of non-malignant MMPs. Heatmap show abundance of MMPs (rows) in each cancer type (columns) for non-malignant cell types, similar to Fig. 4c.

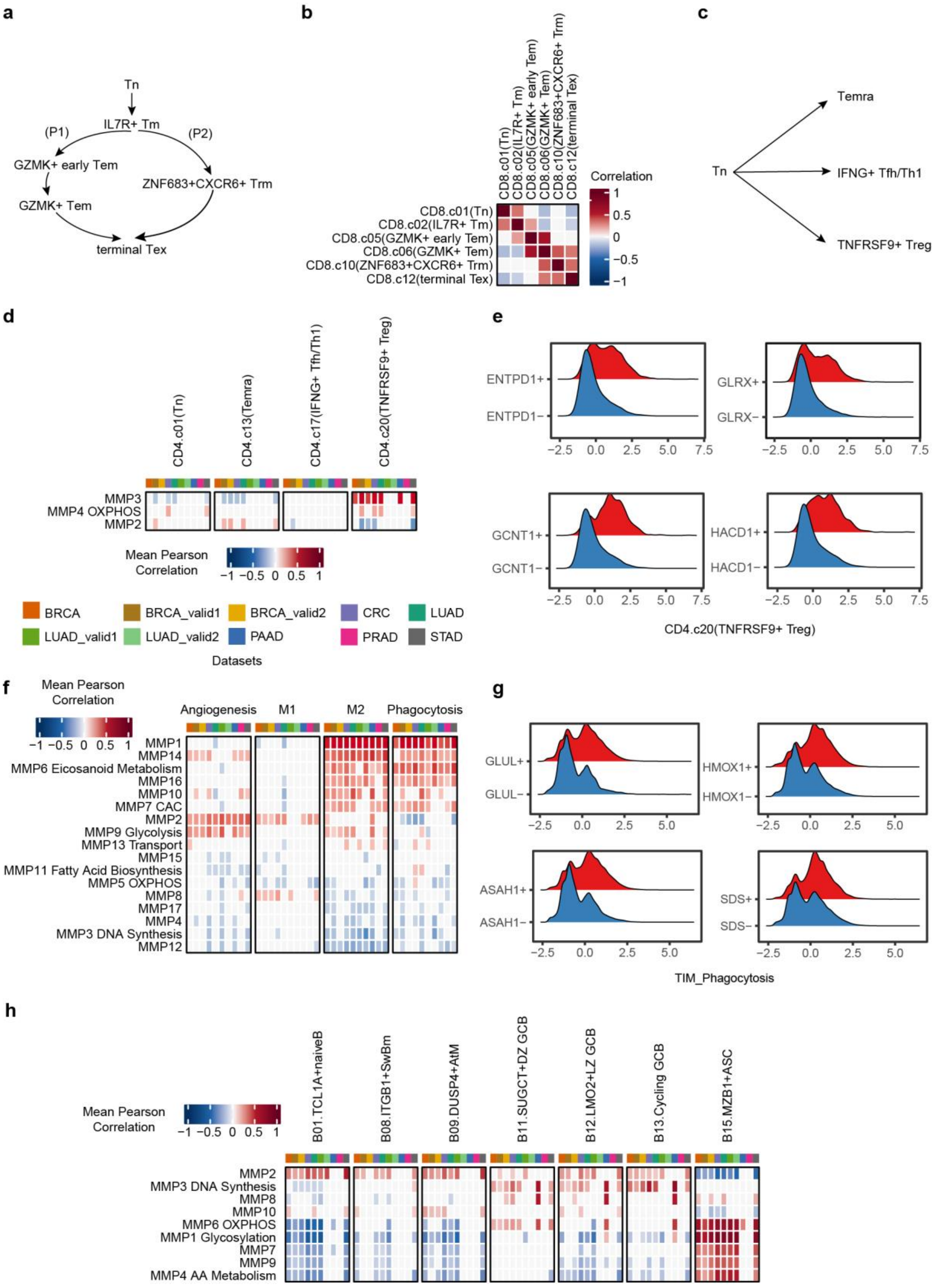
a
Tn
IL7R+ Tm
(P1)
(P2)
GZMK+ early Tem
ZNF683+CXCR6+ Trm
GZMK+ Tem
terminal Tex
b
CD8.c01(Tn)
CD8.c02(IL7R+ Tm)
CD8.c05(GZMK+ early Tem)
CD8.c06(GZMK+ Tem)
CD8.c10(ZNF683+CXCR6+ Trm)
CD8.c12(terminal Tex)
Correlation
c
Tn
Temra
IFNG+ Tfh/Th1
TNFRSF9+ Treg
d
CD4.c01(Tn)
CD4.c13(Temra)
CD4.c17(IFNG+ Tfh/Th1)
CD4.c20(TNFRSF9+ Treg)
MMP3
MMP4 OXPHOS
MMP2
Mean Pearson Correlation
BRCA
BRCA_valid1
BRCA_valid2
CRC
LUAD
LUAD_valid1
LUAD_valid2
PAAD
PRAD
STAD
Datasets
e
ENTPD1+
ENTPD1−
GLRX+
GLRX−
GCNT1+
GCNT1−
HACD1+
HACD1−
CD4.c20(TNFRSF9+ Treg)
f
Mean Pearson Correlation
Angiogenesis
M1
M2
Phagocytosis
MMP1
MMP14
MMP6 Eicosanoid Metabolism
MMP16
MMP10
MMP7 CAC
MMP2
MMP9 Glycolysis
MMP13 Transport
MMP15
MMP11 Fatty Acid Biosynthesis
MMP5 OXPHOS
MMP8
MMP17
MMP4
MMP3 DNA Synthesis
MMP12
g
GLUL+
GLUL−
HMOX1+
HMOX1−
ASAH1+
ASAH1−
SDS+
SDS−
TIM_Phagocytosis
h
Mean Pearson Correlation
B01.TCL1A+naiveB
B08.ITGB1+SwBm
B09.DUSP4+AtM
B11.SUGCT+DZ GCB
B12.LMO2+LZ GCB
B13.Cycling GCB
B15.MZB1+ASC
MMP2
MMP3 DNA Synthesis
MMP8
MMP10
MMP6 OXPHOS
MMP1 Glycosylation
MMP7
MMP9
MMP4 AA Metabolism

Figure S22. MMP associations for immune cells. (a) Illustration for the two paths of T cell exhaustion. T_n, naïve T cells; T_m, memory T cells; T_{em}, effector memory T cells; T_{rm}, tissue-resident memory T cells; T_{ex}, exhausted T cells. **(b)** Correlation heatmap for the signature scores of the above $CD8^+$ T cell metaclusters. The color indicates mean Pearson correlation coefficient as in fig. S14A. **(c)** Illustration for the differentiation of $CD4^+$ T cells. T_{emra}, terminally differentiated effector memory or effector T cells; T_{fh}/T_h1, follicular helper T cell/T helper 1 dual-functional cells; T_{reg}, regulatory T cells. **(d)** Heatmap showing the correlations between MMP scores and signature scores of above $CD4^+$ T cell metacluster, similar plot as in Fig. 6B. **(e)** Ridge plots showing the difference of signature score of the *TNFRSF9*$^+$ T_{reg} metacluster between cells grouped by the expression of representative metabolic genes. **(f)** Heatmap showing the correlations between MMP scores and signature scores of functional phenotypes in macrophages, similar plot as in Fig. 6B. **(g)** Ridge plots showing the difference of signature score of phagocytic macrophages between cells grouped by expression of representative metabolic genes. **(h)** Heatmap showing the correlations between MMP scores and signature scores of B cells subclusters, similar plot as in Fig. 6B.

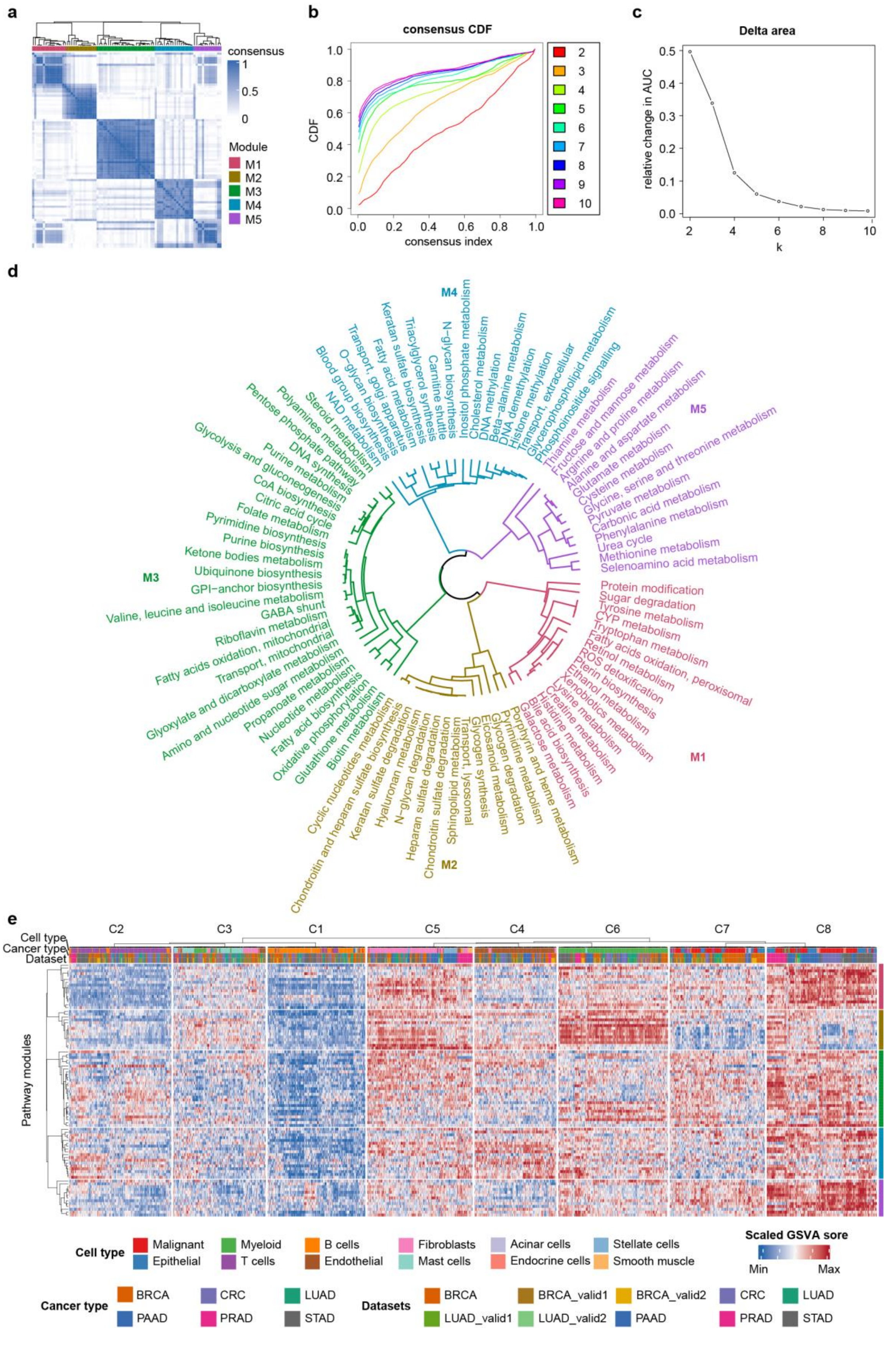
a
consensus
1
0.5
0
Module
M1
M2
M3
M4
M5
b
consensus CDF
CDF
consensus index
c
Delta area
relative change in AUC
k
d
M1
M2
M3
M4
M5
e
C2
C3
C1
C5
C4
C6
C7
C8
Cell type
Cancer type
Dataset
Pathway modules
Cell type
Malignant
Epithelial
Myeloid
T cells
B cells
Endothelial
Fibroblasts
Mast cells
Acinar cells
Endocrine cells
Stellate cells
Smooth muscle
Scaled GSVA sore
Min
Max
Cancer type
BRCA
PAAD
CRC
PRAD
LUAD
STAD
Datasets
BRCA
LUAD_valid1
BRCA_valid1
LUAD_valid2
BRCA_valid2
PAAD
CRC
PRAD
LUAD
STAD

Figure S23. Cluster of pathways and all cell type specific pseudo-bulk samples. (a) Consensus clustering matrix of metabolic pathways for k = 5. **(b)** Plot showing the cumulative distribution function (CDF) curve of the consensus matrix for each k (indicated by colours), estimated by a histogram of 100 bins. **(c)** Delta area plot showing the relative change in area under the CDF curve comparing k and k -1. For k = 2, there is no k -1, so the total area under the curve rather than the relative increase is plotted. **(d)** Hierarchical clustering of metabolic pathways. **(e)** Heatmap showing the clustering results of all cell type specific pseudo-bulk samples based on metabolic pathway scores.

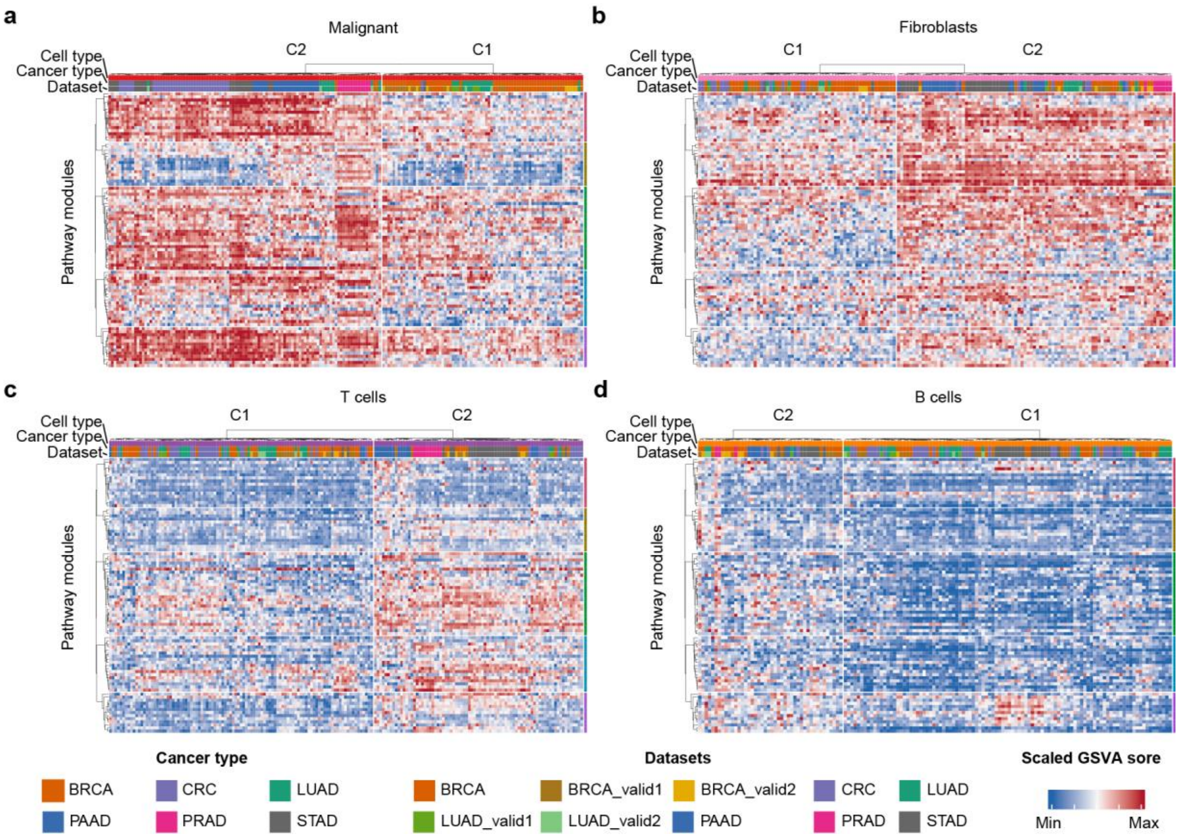

Figure S24. Metabolic subtypes of pan-cancer defined by cellular metabolic properties. Similar plot as in Fig. 7 for other cell types.

Table S1. Metadata for cohorts and samples, provided as an excel file.

Table S2. Canonical markers to assign the cell types, provided as an excel file.

Table S3. Curated metabolic genes and pathways, provided as an excel file.

Table S4. Cell type-specific metabolic properties, provided as an excel file.

Table S5. Cell type-specific metabolic reprogramming, provided as an excel file.

Table S6. MMPs identified in each cell type

Table S7. Number and percent of malignant cells assigned to each MMP, related to Figure 4b, provided as an excel file.

Table S8. shRNA and primer sequence used for knockdown experiments, provided as an excel file.

Table S9. Metabolites significantly changed in knockdown cell lines compared to controls, provided as an excel file.

Table S10. Enriched pathways for Cluster 5, provided as an excel file.
